# Disruption of the Clock Component Bmal1 in Mice Promotes Cancer Metastasis through the PAI‐1‐TGF‐*β*‐myoCAF‐Dependent Mechanism

**DOI:** 10.1002/advs.202301505

**Published:** 2023-06-17

**Authors:** Jieyu Wu, Xu Jing, Qiqiao Du, Xiaoting Sun, Kristian Holgersson, Juan Gao, Xingkang He, Kayoko Hosaka, Chen Zhao, Wei Tao, Garret A. FitzGerald, Yunlong Yang, Lasse D. Jensen, Yihai Cao

**Affiliations:** ^1^ Department of Microbiology, Tumor and Cell Biology Karolinska Institute Stockholm 171 65 Sweden; ^2^ Department of Obstetrics and Gynecology The First Affiliated Hospital Sun Yat‐sen University Zhongshan Second Road 58 Guangzhou 510080 P. R. China; ^3^ Oujiang Laboratory (Zhejiang Lab for Regenerative Medicine, Vison and Brain Health) School of Pharmaceutical Science Wenzhou Medical University Wenzhou 325035 P. R. China; ^4^ Loma Linda University Loma Linda CA 92350 USA; ^5^ Department of Infectious Diseases The Third Affiliated Hospital of Sun Yat‐sen University Guangzhou 510000 P. R. China; ^6^ Department of Gastroenterology Sir Run Run Shaw Hospital Zhejiang University Medical School Hangzhou 310016 P. R. China; ^7^ Eye Institute Eye and ENT Hospital Shanghai Medical College Fudan University Shanghai 200433 P. R. China; ^8^ Center for Nanomedicine and Department of Anesthesiology Brigham and Women's Hospital Harvard Medical School Boston MA 02115 USA; ^9^ Institute for Translational Medicine and Therapeutics University of Pennsylvania Perelman School of Medicine Philadelphia PA 19104‐5158 USA; ^10^ Department of Cellular and Genetic Medicine School of Basic Medical Sciences Fudan University Shanghai 200032 P. R. China; ^11^ Division of Cardiovascular Medicine Department of Medical and Health Sciences Linkoping University Linkoping 581 83 Sweden

**Keywords:** brain and muscle ARNT‐like 1, cancer, circadian clock, fibroblasts, metastasis

## Abstract

The circadian clock in animals and humans plays crucial roles in multiple physiological processes. Disruption of circadian homeostasis causes detrimental effects. Here, it is demonstrated that the disruption of the circadian rhythm by genetic deletion of mouse brain and muscle ARNT‐like 1 (Bmal1) gene, coding for the key clock transcription factor, augments an exacerbated fibrotic phenotype in various tumors. Accretion of cancer‐associated fibroblasts (CAFs), especially the alpha smooth muscle actin positive myoCAFs, accelerates tumor growth rates and metastatic potentials. Mechanistically, deletion of Bmal1 abrogates expression of its transcriptionally targeted plasminogen activator inhibitor‐1 (PAI‐1). Consequently, decreased levels of PAI‐1 in the tumor microenvironment instigate plasmin activation through upregulation of tissue plasminogen activator and urokinase plasminogen activator. The activated plasmin converts latent TGF‐*β* into its activated form, which potently induces tumor fibrosis and the transition of CAFs into myoCAFs, the latter promoting cancer metastasis. Pharmacological inhibition of the TGF‐*β* signaling largely ablates the metastatic potentials of colorectal cancer, pancreatic ductal adenocarcinoma, and hepatocellular carcinoma. Together, these data provide novel mechanistic insights into disruption of the circadian clock in tumor growth and metastasis. It is reasonably speculated that normalization of the circadian rhythm in patients provides a novel paradigm for cancer therapy.

## Graphical abstract

Compared to non‐disrupted (left), disruption of the circadian rhythm such as deletion of Bmal1 gene results in PAI‐1 reduction (right), elevated activation of plasmin, and subsequently high levels of the mature form of TGF‐*β*. TGF‐*β* is a potent fibrotic factor to cancer fibrosis and CAFs‐myoCAFs conversion. Expansion of CAF in the TME increases tumor mass and facilitates metastasis.

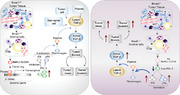

## 1 Introduction

Circadian clocks as endogenous oscillators with a 24 h cycle each day control numerous physiological processes, including metabolism, sleep, immunity, neurological activity, endocrinology, and vascular biology.^[^ ^1^ ^]^ Now, we know that specialized clock genes at the transcriptional level control circadian rhythms and behaviors, and these genes are highly conserved across all species.^[^ ^1^, ^2^, ^3^, ^4^ ^]^ Brain and muscle ARNT‐like 1 (Bmal1; MOP3 or ARNTL) is an indispensable transcription component that heterodimerizes with CLOCK to activate a myriad of its targeted and circadian regulated genes.^[^ ^3^, ^4^, ^5^, ^6^ ^]^ In mammals, disruption of Bmal1 instigates a broad range of abnormalities, including eradication of circadian rhythms, neurodegeneration, visual impairment, metabolic dysfunction, oxidative stress, and shortened lifespan.^[^ ^2^, ^3^, ^4^, ^5^, ^6^, ^7^, ^8^, ^9^ ^]^ Some of these pathophysiological roles of Bmal1 might contribute to tumor growth and metastasis.^[^ ^10^, ^11^ ^]^ Despite this known knowledge, the role of circadian rhythms in various pathological conditions are, however, less understood. Disruption of the circadian clock has been linked to high cancer incidences and accelerated tumor growth rates.^[^ ^12^, ^13^ ^]^ Disruption of circadian rhythms in cancer cells in promoting metastasis has recently been reported.^[^ ^14^ ^]^ For example, BAML1 promotes human cancer cell proliferation, migration, survivals, and invasion,^[^ ^15^, ^16^, ^17^, ^18^ ^]^ which may involve interactions between cancer cells and stromal cell interaction in the tumor microenvironment (TME).^[^ ^19^, ^20^, ^21^, ^22^, ^23^ ^]^ However, the impact of circadian disruption in cancer hosts and TME on metastasis remains unexplored.

A solid tumor tissue contains genetically altered malignant cells and various stromal cellular components, including stromal fibroblasts, inflammatory cells, immune cells, vascular cells, and adipocytes.^[^ ^3^, ^24^, ^25^, ^26^ ^]^ Along cancer progression, the composition and phenotype of stromal cellular components constantly alters to support tumor growth and metastasis. In some cancers, such as pancreatic ductal adenocarcinoma (PDAC), the stromal components constitute over 90% of the entire tumor mass and the ratio of stromal fibrotic component versus cancer cells correlates with poor prognosis.^[^ ^27^, ^28^ ^]^ Further, inflammation, fibrosis, and angiogenesis in the TME play causative roles in promoting cancer invasion, metastasis, and drug resistance.^[^ ^29^, ^30^, ^31^, ^32^, ^33^, ^34^ ^]^ Thus, targeting tumor stromal components provides effective approaches for cancer therapy. In fact, current anticancer drugs that target stromal components, including antiangiogenic drugs, immunotherapeutics, and anti‐inflammatory drugs seem to be more effective than those drugs designed to directly target cancer cells.^[^ ^26^ ^]^ Moreover, drugs targeting tumor stromal components provide generalized approaches for treating various types of cancer.

Metastasis is the leading cause of cancer‐related mortality and can occur when a primary tumor is tiny.^[^ ^35^ ^]^ The metastatic cascade includes several concrete steps, including cancer cell invasion at the primary site, intravasation into the circulation, transport to distal tissues and organs via bloodstream or lymphatics, extravasation, formation of the initial metastatic niche in distal tissues, and regrowth to a clinically detectable metastatic mass.^[^ ^36^, ^37^, ^38^ ^]^ In the TME, cancer‐associated fibroblasts (CAFs) have intertwined interactions with cancer cells and promote metastasis at different steps.^[^ ^33^, ^39^, ^40^ ^]^ CAFs form complex clusters with cancer cells and hijack them for intravasation and possibly extravasation.^[^ ^41^ ^]^ In the distal organ, CAFs may participate in the formation of the initial metastatic niches and serve as feeders to facilitate the growth of metastases.^[^ ^42^ ^]^ In particular, the alpha smooth muscle actin positive (*α*‐SMA^+^) CAF subpopulation could potentially facilitate the carriage of cancer cells to various organs.^[^ ^28^, ^43^ ^]^

Plasminogen activator inhibitor‐1 (PAI‐1) is a serine protease inhibitor that serves as the key inhibitor of tissue plasminogen activator (tPA) and urokinase plasminogen activator (uPA), two main activators of converting plasminogen into plasmin.^[^ ^44^ ^]^ Transforming growth factor beta (TGF‐*β*) is a multifunctional cytokine family composing of TGF‐*β*1, TGF‐*β*2, TGF‐*β*3, and many other related signaling molecules.^[^ ^45^ ^]^ TGF‐*β* is synthesized in various cell types as its latent form by forming a protein complex with latent TGF‐*β* binding protein and latency‐associated peptide.^[^ ^46^ ^]^ Serum proteinases such as plasmin catalyze the conversion of the latent TGF‐*β* into its active form. Activated TGF‐*β* together with several other factors form a serine/threonine kinase complex to bind TGF‐*β* receptors, which are composed of type 1 and type 2 receptor subunits.^[^ ^47^ ^]^ After TGF‐*β* stimulation, the type 2 receptor kinase activates the type 1 receptor kinase by phosphorylation that subsequently triggers a signaling cascade, including differentiation, chemotaxis, and proliferation of the targeted cells.^[^ ^48^ ^]^ Fibroblasts are one of the main targeted cell types by TGF‐*β*.^[^ ^49^ ^]^ Because the proteolytic cleavage for releasing the activated TGF‐*β* is a rate‐limiting and temporospatial process, controlling activation of plasmin in a local environment is crucial for regulating TGF‐*β* functions.

In this study, we report that the circadian clock gene Bmal1 transcriptionally targets PAI‐1 to suppress TGF‐*β* functions by inhibiting plasmin production. Genetic deletion of Bmal1 in mice reduced PAI‐1 expression in the TME, leading to plasmin activation through upregulation of tPA and uPA. Subsequently, plasmin activated TGF‐*β* to induce tumor fibrosis and the CAF‐myoCAF transition, which collectively promoted tumor growth and metastasis. Pharmacological inhibition of the TGF‐*β* signaling largely ablated the circadian disruption–triggered cancer metastasis. Taken together, our data provide novel mechanistic insights into how disruption of circadian rhythms in relation to metastasis and propose a new paradigm for cancer therapy.

## 2 Results

### 2.1 Accelerated Tumor Growth Rates and Hyperfibrosis in Bmal1^−/−^ Mice

**Figure 1:**
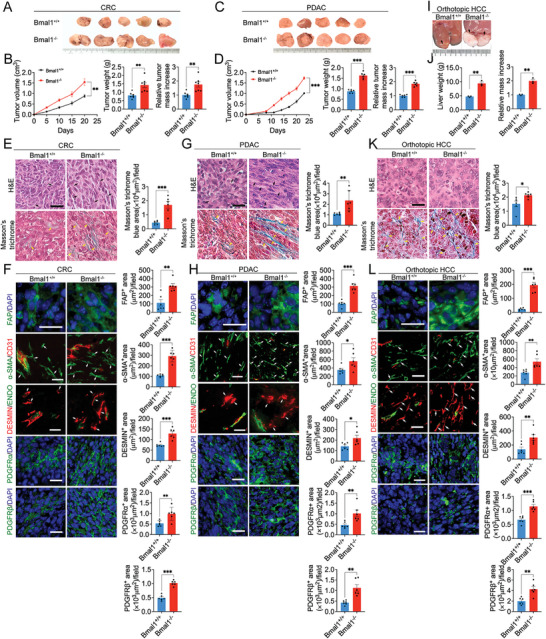
Genetic deletion of Bmal1 in mice promotes tumor growth and fibrosis. A) Representative picture of subcutaneous mouse CRC tumors grown in Bmal1^+/+^ and Bmal1^−/−^ mice. B) Quantification of CRC tumor growth rates, tumor weight, and tumor relative mass increase in Bmal1^+/+^ and Bmal1^−/−^ mice (*n* = 7 mice per group). C) Representative picture of subcutaneous PDAC tumors grown in Bmal1^+/+^ and Bmal1^−/−^ mice. D) Quantification of PDAC tumor growth rates, tumor weights, and tumor relative mass increase in Bmal1^+/+^ and Bmal1^−/−^ mice (*n* = 6 mice per group). E) H&E and Masson's trichrome staining of CRC tumors (*n* = 6 random fields per group). F) Representative micrographs and quantification of immunofluorescence staining of CRC tumor tissues in Bmal1^+/+^ and Bmal1^−/−^ mice with fibroblast markers: FAP (green); *α*‐SMA (green) plus CD31 (red); DESMIN (red) plus ENDOMUCIN (green); PDGFR*α* (green); and PDGFR*β* (green) (*n* = 6 random fields per group). G) H&E and Masson's trichrome staining of PDAC tumors in Bmal1^+/+^ and Bmal1^−/−^ mice (*n* = 6 random fields per group). H) Representative micrographs and quantification of immunofluorescence staining of PDAC tumor tissues with fibroblast markers: FAP (green); *α*‐SMA (green) plus CD31 (red); DESMIN (red) plus ENDOMUCIN (green); PDGFR*α* (green); and PDGFR*β* (green) (*n* = 6 random fields per group). I) Representative pictures of orthotopic HCC tumors grown in Bmal1^+/+^ and Bmal1^−/−^ mice. J) Quantification of orthotopic HCC tumor, liver weights, and relative liver mass increase in Bmal1^+/+^ and Bmal1^−/−^ mice (*n* = 3 mice per group). K) H&E and Masson's trichrome staining of orthotopic HCC tumors in Bmal1^+/+^ and Bmal1^−/−^ mice (*n* = 6 random fields per group). L) Representative micrographs and quantification of immunofluorescence staining of HCC tumor tissues with fibroblast markers: FAP (green); *α*‐SMA (green) plus CD31 (red); DESMIN (red) plus ENDOMUCIN (green); PDGFR*α* (green); and PDGFR*β* (green) (*n* = 6 random fields per group). Data presented as mean ± S.E.M. *P < 0.05; **P < 0.01; ***P < 0.001; two‐tailed student *t*‐test. Scale bar = 50 µm.

To study the impact of circadian rhythms on tumor growth, murine colorectal cancers (CRC) were subcutaneously implanted into Bmal1 wild type (Bmal1^+/+^) and knockout (Bmal1^−/−^) mice. Interestingly, CRC tumors showed an accelerated growth rate in Bmal1^−/−^ mice relative to those grown in Bmal1^+/+^ mice (**Figure** **1**A,B). By day 20 after tumor implantation, an ≈70% increase of tumor mass was detected in Bmal1^−/−^ mice (Figure 1A,B). To validate these findings, murine PDAC tumor cells were implanted in both Bmal1^+/+^ and Bmal1^−/−^ mice. Similar to the CRC model, PDAC exhibited an accelerated growth rate in Bmal1^−/−^ mice (Figure 1C,D). These data show that deletion of Bmal1 in mice results in accelerated tumor growth rates.

Histological examination of CRC tumors discovered high contents of Masson's trichrome positive fibrotic components in tumor tissues grown in Bmal1^−/−^ mice relative to Bmal1^+/+^ animals (Figure 1E). Immunohistochemical analysis demonstrated that CRC tumors in Bmal1^−/−^ mice contained increased stromal fibroblasts, including positive structures of fibroblast activation protein (FAP), *α*‐SMA, DESMIN, platelet‐derived growth factor receptor alpha (PDGFR*α*), and platelet‐derived growth factor receptor beta (PDGFR*β*) (Figure 1F). Similarly, Masson's trichrome, FAP, *α*‐SMA, DESMIN, PDGFR*α*, and PDGFR*β* positive fibrotic structures were also markedly elevated in PDAC tumors grown Bmal1^−/−^ mice (Figure 1G,H). Quantitative analysis by quantitative polymerase chain reaction (qPCR) further validated high mRNA expression levels of these fibrotic markers in CRC and PDAC tumors (Figure S1, Supporting Information).

In addition to subcutaneous CRC and PDAC models, we further employed an orthotopic cancer model of murine hepatocellular carcinoma (HCC) in our experimental settings. Mouse HCC tumors also grew at an accelerated rate in Bmal1^−/−^ mice relative to Bmal1^+/+^ animals (Figure 1I,J). HCC tumors also contained high fibrotic components in Bmal1^−/−^ mice (Figure 1K,L and Figure S1, Supporting Information). Together, these results demonstrate that genetic deletion of Bmal1 gene in mice leads to accelerated tumor growth rates and increased fibrotic components.

### 2.2 The Impact of Bmal1 on CAF Proliferation and Tumor Microenvironment

We next studied cell proliferation and alteration of the TME in tumors grown in Bmal1^+/+^ and Bmal1^−/−^ mice. Both CRC and PDAC tumor cells were labeled green fluorescent protein (GFP). Nuclear protein Ki67 (Ki67) staining showed significant increases of proliferating cell populations in CRC tumors grown in Bmal1^−/−^ mice (Figure S2A,B, Supporting Information). Co‐immunostaining revealed a marked increase of Ki67^+^/FAP^+^ double‐positive cell population (Figure S2A,B, Supporting Information), indicating an elevated fibrosis in tumors grown in Bmal1^−/−^ mice. Consequently, the total cell numbers indicated by 4′,6‐diamidino‐2‐phenylindole (DAPI) were markedly increased (Figure S2A,B, Supporting Information). Similar results were also obtained from the PDAC and HCC models (Figure S3, Supporting Information). Interestingly, the Ki67/GFP double positive CRC and PDAC tumor cells were not significantly increased (Figures S2A,B and S3A,B, Supporting Information). These data are consistent with increases of CAF components mainly accounting for the accelerated tumor growth rates in Bmal1^−/−^ mice.

In contrast to increases of proliferating cells, apoptotic cell numbers were significantly decreased in CRC, PDAC, and HCC tumors grown in the Bmal1^−/−^ background (Figures S2 and S3, Supporting Information). Interestingly, Cluster of differentiation 31 (CD31^+^) microvascular density was also significantly increased. However, these vessels lacked sufficient coverage of NG2^+^ perivascular cells. Mitigation of cellular apoptosis and increases of microvessels further supported the elevated tumor growth rates in Bmal1^−/−^ animals. Additionally, tumors in Bmal1^−/−^ mice experienced more hypoxia by expressing high levels of CA9 relative to those grown in Bmal1^+/+^ mice (Figures S2 and S3, Supporting Information). Although F4/80^+^ tumor‐associated macrophages (TAMs) were slightly decreased in tumors grown in Bmal1^−/−^ mice. Since the M2 subtype, but the M1 subtype, of TAMs is involved in tumor invasion and metastasis, ^[^ ^28^ ^]^ we further defined M1 and M2 TAM subpopulations using their specific markers. The Cluster of differentiation 206 (CD206^+^) M2 population was not altered (Figures S2 and S3, Supporting Information). Similarly, the CD80^+^ M1 subpopulation of TAMs also remained unchanged (Figures S2 and S3, Supporting Information).

### 2.3 Mitigation of PAI‐1 in Tumor Stromal Cells

**Figure 2:**
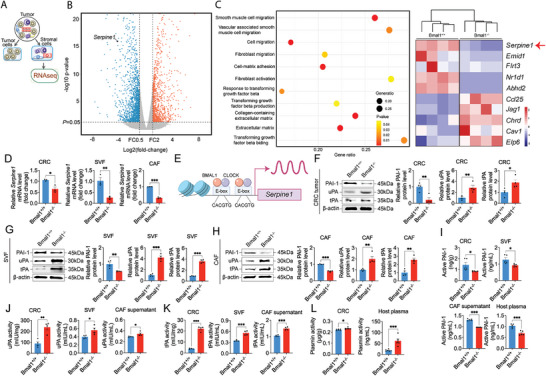
Bmal1 deficiency augments the PAI‐1‐tPA/uPA‐plasmin axis. A) The stromal vascular fraction (SVF) of CRC tumors grown in Bmal1^+/+^ and Bmal1^−/−^ mice were isolated by FACS sorting. RNAs were extracted and subjected for sequencing. B) Volcano plot presenting differential gene expression in isolated SVF from Bmal1^+/+^ versus Bmal1^−/−^ mice. Orange indicates significantly increased genes and blue indicates significantly decreased genes. C) Genome‐wide expression profiling of SVF from Bmal1^+/+^ and Bmal1^−/−^ tumor tissues (*n* = 4 samples per group). Serpine1 was identified as one of the highly down regulated genes. D) Quantification of Serpine1 mRNA level in CRC tumors, SVF, and FACS‐isolated FAP^+^ CAFs from Bmal1^+/+^ and Bmal1^−/−^ tumors (*n* = 3 samples per group). E) Schematic diagram of Serpine1 transcription. The Serpine1 is a direct transcriptional target of BMAL1‐CLOCK heterodimers and the Serpine1 gene promoter contained canonical E‐boxes of BMAL1‐CLOCK binding sites. F–H) Relative PAI‐1, uPA, and tPA protein levels and quantification in CRC tumors, SVF, and CAFs from Bmal1^+/+^ and Bmal1^−/−^ mice (*n* = 4 samples per group). I) ELISA quantification of active PAI‐1 in CRC tumors, SVF, CAF supernatant and plasma from tumor‐bearing mice (*n* = 5 samples per group). J,K) Quantification of uPA and tPA activity in CRC tumors, SVF, and CAF supernatant from Bmal1^+/+^ and Bmal1^−/−^ mice (*n* = 4 samples per group). L) Quantification of plasmin activity in CRC tumor and plasma from tumor‐bearing mice (*n* = 6 samples per group). Data presented as mean ± S.E.M. *P < 0.05; **P < 0.01; ***P < 0.001; two‐tailed student *t*‐test.

Because the Bmal1 gene was deleted in cancer host, but not in cancer cells per se, we next isolated Bmal1^−/−^ tumor stromal cells by fluorescence‐activated single cell sorting (FACS) to explore the key molecular signaling in the stromal components (**Figure** **2**A). We used FACS to sort out the CRC tumor cells that were labeled with strong GFP signals and isolated the stromal components. The GFP labeled and non‐labeled tumor cells were applied to set the sorting gate. The isolated Bmal1^−/−^ stromal cells, containing CAFs, TAMs, vascular cells, and other immune cells were subjected to RNA sequencing. Serpine1, the gene coding for PAI‐1, was one of the most markedly down‐regulated genes in the stromal cells of CRCs grown in Bmal1^−/−^ mice (Figure 2B). Bioinformatic analysis by gene ontology (GO) enrichment targeting fibrotic related contents showed that fibrosis in tumors grown in Bmal1^−/−^ mice was markedly altered (Figure 2B). Interestingly, Serpine1 was the most downregulated gene in the stromal fraction of CRC tumors grown in the Bmal1^−/−^ background (Figure 2C). These bioinformatic findings are consistent with accelerated fibrosis in CRC tumors grown in Bmal1^−/−^ mice.

qPCR analysis further validates the detection of Serpine1 in CRC tumors grown in Bmal1^−/−^ mice relative to Bmal1^+/+^ mice (Figure 2D). To further delineate the cellular source of Serpine1 downregulation, CRC cancer cells and stromal cells were FACS sorted from CRC tumors. Stromal cellular components of the stromal vascular fraction (SVF) and FAP^+^CAFs, but not tumor cells, exhibited marked downregulation of Serpine1 mRNA levels (Figure 2D and Figure S4, Supporting Information), corroborating the stromal source of Serpine1 downregulation. Analysis of the Serpine1 gene promoter identified two classical E‐boxes for Bmal1 binding (Figure 2E).^[^ ^50^ ^]^ Consistent with downregulation of mRNA levels of Serpine1, immunoblot analysis demonstrated downregulation of PAI‐1 protein (Figure 2F). Since PAI‐1 is the key inhibitor for tPA and uPA, we next analyze tPA and uPA in our experimental settings. Protein levels of tPA and uPA were increased in tumor tissues grown in Bmal1^−/−^ mice (Figure 2F). Downregulation of PAI‐1 and upregulation of tPA and uPA proteins were further validated in SVFs and CAFs of CRC tumors grown in Bmal1^−/−^ mice (Figure 2G,H).

Next, we analyzed PAI‐1, uPA, and tPA activities in the tumor tissues, isolated SVFs and CAFs. While active PAI‐1 was marked decreased in CRC tumors grown in Bmal1^−/−^ mice (Figure 2I), uPA and tPA activities were substantially increased (Figure 2J,K). Similar downregulation of active PAI‐1 was also observed in the plasma of CRC tumor‐bearing Bmal1^−/−^ mice (Figure 2I). Along with alterations of PAI‐1, tPA, and uPA activities, plasmin activities in tumors and plasma were accordingly increased in Bmal1^−/−^ mice (Figure 2L). Together, these findings show that PAI‐1 was markedly mitigated in the stromal compartment of tumors grown in Bmal1^−/−^ mice. The reduction of PAI‐1 results in activation of tPA and uPA, which subsequently activate plasmin.

### 2.4 Activation of the TGF‐*β* Signaling

**Figure 3:**
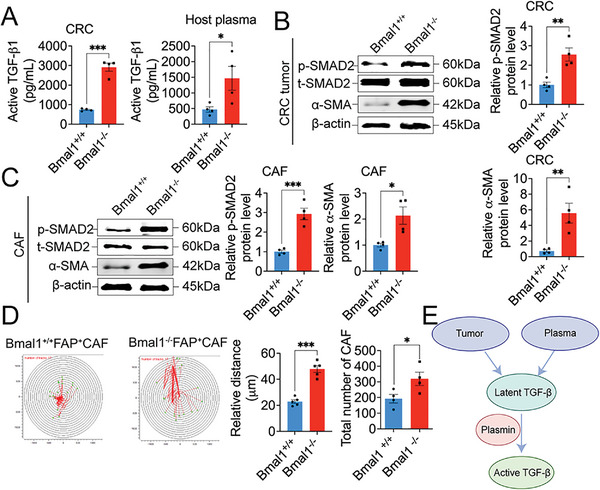
Activation of TGF‐*β* signaling in Bmal1^−/−^ tumor and CAFs. A) ELISA quantification of active TGF‐*β* in CRC tumors and plasma from tumor‐bearing mice (*n* = 4 samples per group). B,C) Immunoblot and quantification of p‐SMAD2 and *α*‐SMA in CRC tumor and CAFs from Bmal1^+/+^ and Bmal1^−/−^ mice (*n* = 4 samples per group). D) Cell motility and quantification (*n* = 5 colonies per group). Quantification of CAFs number after 48 h from Bmal1^+/+^ and Bmal1^−/−^ mice (*n* = 4 random fields per group). E) Schematic diagram of activation of TGF‐*β* signaling. Tumor and tumor‐bearing host plasma provide resource of latent TGF‐*β*. Bmal1 deficiency augment PAI‐1‐uPA/tPA‐plasmin axis. High plasmin serves as activator convert latent TGF‐*β* into active form. Data presented as mean ± S.E.M. *P < 0.05; **P < 0.01; ***P < 0.001; two‐tailed student *t*‐test.

Since plasmin is a key activator for the TGF‐*β* signaling by proteolytically converting the latent TGF‐*β* into the active form of TGF‐*β*, we next investigated the activation of TGF‐*β* signaling in tumors grown in Bmal1^+/+^ and Bmal1^−/−^ mice. The active form of TGF‐*β* in tumors and plasma of Bmal1^−/−^ mice was markedly increased (**Figure** **3**A). Consistent with the increase of TGF‐*β* activity, mothers against decapentaplegic homolog 2 (SMAD2), one of the key downstream signaling components of TGF‐*β* signaling,^[^ ^51^ ^]^ became activated by phosphorylation (Figure 3B). The *α*‐SMA positive signals also increased in tumors grown in Bmal1^−/−^ mice (Figure 3B). Similarly, levels of phosphorylated SMAD2 and *α*‐SMA were also markedly increased in isolated CAFs (Figure 3C). We next studied CAF migration by tracing cell motility owing to the following reasons: 1) TGF‐*β* is a potent growth factor for fibroblasts by stimulating cell proliferation and migration; 2) *α*‐SMA positive signals that represented myofibroblasts are markedly increased in Bmal1^−/−^ CAFs, which should be highly motile; 3) CAF motility is highly associated with cancer metastasis.^[^ ^28^ ^]^ Expectedly, Bmal1^−/−^ CAFs exhibited increased motility relative to Bmal1^+/+^ CAFs (Figure 3D), supporting a myofibroblast phenotype. Together, these results show that tumor‐ and plasma‐derived latent TGF‐*β* was converted into active TGF‐*β* by increased plasmin activity in Bmal1^−/−^ mice (Figure 3E).

### 2.5 Inhibition of TGF‐*β* Ablates Accelerated Tumor Growth Rates in Bmal1^−/−^ Mice by Suppression of Fibrosis

**Figure 4:**
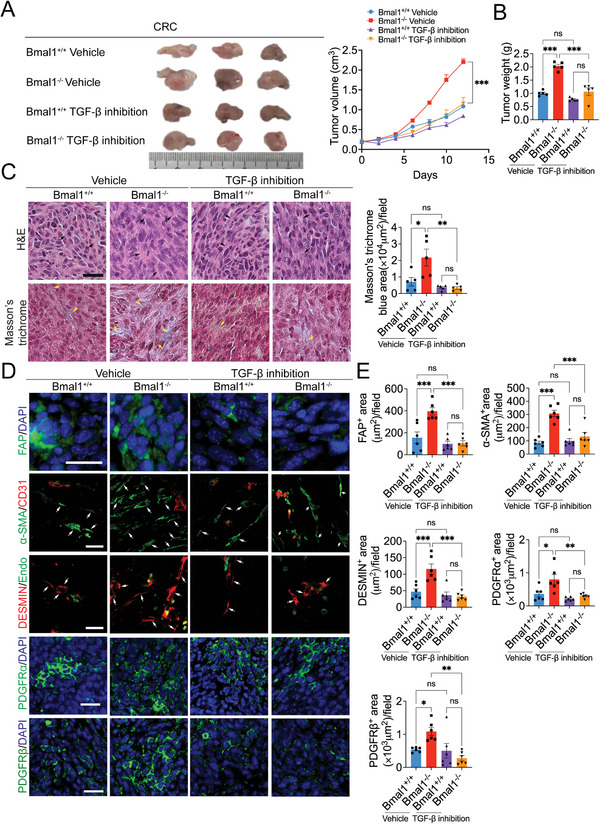
Ablation of Bmal1 deletion‐accelerated tumor growth and tumor fibrosis by inhibition of TGF‐*β*. A) Representative pictures (left) and growth rate (right) of CRC tumor treated with vehicle and SB‐431542 grown in Bmal1^+/+^ and Bmal1^−/−^ mice (*n* = 5 mice per group). B) Quantification of tumor weight of vehicle‐ and SB‐431542‐treated CRC tumors in Bmal1^+/+^ and Bmal1^−/−^ mice (*n* = 5 mice per group). C) H&E and Masson's trichrome staining of vehicle‐ and SB‐431542‐treated CRC tumors in Bmal1^+/+^ and Bmal1^−/−^ mice (*n* = 5 random fields per group). D,E) Representative micrographs and quantification of immunofluorescence staining of vehicle‐ and SB‐431542‐treated CRC tumor tissues in Bmal1^+/+^ and Bmal1^−/−^ mice with fibroblast markers: FAP (green); *α*‐SMA (green) plus CD31 (red); DESMIN (red) plus ENDOMUCIN (green); PDGFR*α* (green); and PDGFR*β* (green) (*n* = 6 random fields per group). Data presented as mean ± S.E.M. *P < 0.05; **P < 0.01; ***P < 0.001, ns = not significant; one‐way ANOVA. Scale bar = 50 µm.

To study the impact of TGF‐*β* on tumor growth and fibrosis in Bmal1^−/−^ mice, a TGFbR1 inhibitor, SB‐431542,^[^ ^52^ ^]^ was employed in our study. Treatment of CRC tumors with SB‐431542 in Bmal1^+/+^ mice did not affect tumor growth (**Figure** **4**A,B). By contrast, inhibition of transforming growth factor‐beta receptor 1 (TGF‐*β*R1) in Bmal1^−/−^ mice produced a markedly inhibitory effect on tumor growth, which was indistinguishable from the vehicle‐treated control tumors grown in Bmal1^+/+^ mice (Figure 4A,B). These results show that accelerated tumor growth rates in Bmal1^−/−^ mice are mediated through a TGF‐*β*‐dependent mechanism.

Consistent with tumor suppression, pharmacological inhibition of TGF‐*β*R1 abrogated Bmal1‐deletion–triggered tumor fibrosis that showed reduced positive structures of FAP, *α*‐SMA, DESMIN, PDGFR*α*, and PDGFR*β* (Figure 4C–E). The levels of these fibrotic components were indistinguishable from the vehicle‐treated control tumors grown in Bmal1^+/+^ mice (Figure 4E and Figure S5, Supporting Information). These data are consistent with TGF‐*β* elevating tumor fibrosis in Bmal1^−/−^ mice. Inhibition of TGF‐*β*R1 by SB‐431542 also significantly suppressed tumor cell proliferation (Figure S6A,B, Supporting Information). Importantly, inhibition of the Ki67^+^ cell population by SB‐431542 was restricted to the FAP^+^ cells, but not GFP^+^ cancer cells (Figure S6A,B, Supporting Information). Other cellular components in the TME, including CD31^+^ and F4/80^+^ structures remained unchanged in response to TGF‐*β*R1 in Bmal1^−/−^ mice (Figure S6A,B, Supporting Information). Together, these data are consistent with the finding that TGF‐*β* is responsible for tumor fibrosis and accelerated tumor growth in Bmal1^−/−^ mice.

### 2.6 TGF‐*β*‐Dependent Activation of CAFs

**Figure 5:**
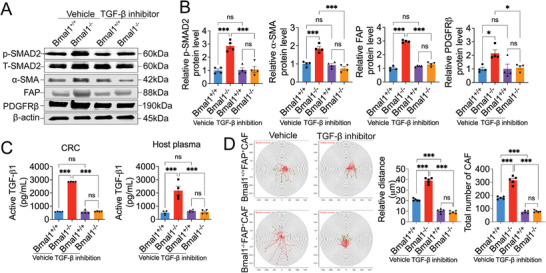
TGF‐*β* mediates myoCAF transition in Bmal1^−/−^ tumors. A) Immunoblot analysis of p‐SMAD2, t‐SMAD2, *α*‐SMA, FAP, and PDGFR*β* of vehicle‐ and SB‐431542‐treated tumors grown in Bmal1^+/+^ and Bmal1^−/−^ mice. B) Quantification of protein levels of p‐SMAD2, *α*‐SMA, FAP, and PDGFR*β* in Bmal1^+/+^ and Bmal1^−/−^ mice (*n* = 4 samples per group). C) ELISA quantification of protein levels of active TGF‐*β* in vehicle‐ and SB‐431542‐treated tumors and plasma of tumor‐bearing mice (*n* = 4 samples per group). D) Cell motility and quantification of FACS‐isolated FAP^+^ CAFs from vehicle‐ and SB‐431542‐treated Bmal1^+/+^ and Bmal1^−/−^ tumors (*n* = 5 colonies per group). Quantification of CAF number after 48 h (*n* = 5 random fields per group). Data presented as mean ± S.E.M. ***P < 0.001, ns = not significant; one‐way ANOVA.

To provide further experimental evidence of TGF‐*β* in activation of CAFs, SB‐431542‐treated tissues were quantitatively analyzed for CAF markers. In vehicle‐treated and SB‐431542 treated Bmal1^+/+^CRC tumor–bearing mice, there was no elevated expression of p‐Smad2 (**Figure** **5**A,B). In consistent with TGF‐*β* activation in tumors grown in Bmal1^−/−^ mice, an elevated level of p‐Smad2 was detected. Intriguingly, inhibition of TGF‐*β*R1 abolished the elevated expression of p‐Smad2 (Figure 5A,B). Consistent with TGF‐*β*‐p‐Smad2 activation, *α*‐SMA, FAP, and PDGFR*β* expression in tumors grown in Bmal1^−/−^ mice was markedly increased (Figure 5A,B), suggesting that TGF‐*β* instigated the CAF‐to‐myoCAF transition. Again, SB‐431542 completely abrogated *α*‐SMA, FAP, and PDGFR*β* expression (Figure 5A,B). The active form of TGF‐*β* was markedly increased in tumor and plasma of Bmal1^−/−^ mice. However, in response to inhibition of TGF‐*β*R1, active TGF‐*β* markedly decreased in tumors and plasma of Bmal1^−/−^ mice (Figure 5C).

We next isolated CAFs from Bmal1^+/+^ and Bmal1^−/−^ mice, and performed a cell motility assay. Genetic deletion of Bmal1^−/−^ markedly increased CAF motility relative to Bmal1^+/+^‐CAFs (Figure 5D). However, Bmal1^−/−^ CAF motility was completely blocked by TGF‐*β*R1 inhibition (Figure 5D). These findings indicate that TGF‐*β* instigates the CAF‐myoCAF transition and cell motility in Bmal1^−/−^ mice.

### 2.7 Genetic Deletion of Bmal1 Promotes Cancer Metastasis

**Figure 6:**
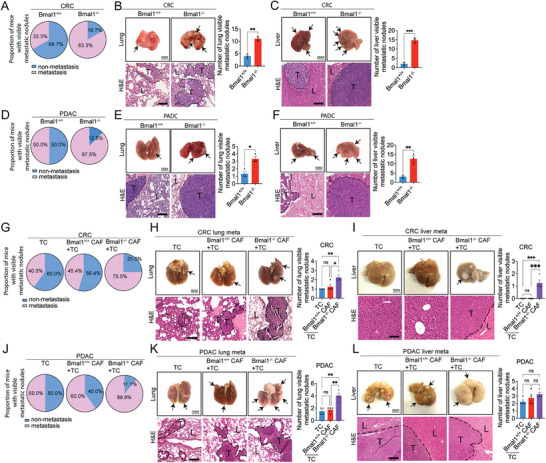
Genetic deletion of Bmal1 promotes cancer metastasis. A) Quantification of visible metastases on the surface of lungs and livers in CRC tumor‐bearing Bmal1^+/+^ and Bmal1^−/−^ mice (*n* = 6 mice per group). B,C) Gross examination and H&E staining of lungs and livers of CRC tumor‐bearing Bmal1^+/+^ and Bmal1^−/−^ mice (scale bar = 5 mm). Quantification of visible metastatic nodules by H&E staining (*n* = 3 random fields per group). D) Quantification of visible metastases on the surface of lungs and livers in PDAC tumor‐bearing Bmal1^+/+^ and Bmal1^−/−^ mice (*n* = 8 mice per group). E,F) Gross examination and H&E staining of lungs and livers of PDAC tumor‐bearing Bmal1^+/+^ and Bmal1^−/−^ mice (scale bar = 5 mm). Quantification of visible metastatic nodules by H&E staining (*n* = 3 random fields per group). G) Quantification of visible metastases on the surface of lungs and livers in C57BL/6 wild type mice that received CRC tumor cells, CRC tumor cells plus Bmal1^+/+^ CAFs, and CRC tumor cells plus Bmal1^−/−^ CAFs implantation (*n* = 10 mice per group). H,I) Gross examination and H&E staining of lungs and livers of CRC tumor‐bearing in C57BL/6 wild type mice that received CRC tumor cells, CRC tumor cells plus Bmal1^+/+^ CAFs, and CRC tumor cells plus Bmal1^−/−^ CAFs implantation (scale bar = 5 mm). Quantification of visible metastatic nodules by H&E staining (*n* = 4 random fields per group). J) Quantification of visible metastases on the surface of lungs and livers in PDAC tumor‐bearing in C57BL/6 wild type mice that received PDAC tumor cells, PDAC tumor cells plus Bmal1^+/+^ CAFs, and PDAC tumors cell plus Bmal1^−/−^ CAFs implantation (*n* = 10 mice per group). K,L) Gross examination and H&E staining of lungs and livers of PDAC tumor‐bearing in C57BL/6 wild type mice that received PDAC tumor cells, PDAC tumor cells plus Bmal1^+/+^ CAFs, and PDAC tumor cells plus Bmal1^−/−^ CAFs implantation (scale bar = 5 mm). Quantification of visible metastatic nodules by H&E staining (*n* = 4 random fields per group). Data presented as mean ± S.E.M. *P < 0.05; **P < 0.01; ***P < 0.001, ns = not significant; two‐tailed student *t*‐test and one‐way ANOVA. TC = tumor cell. Apparent metastatic nodules are indicated by arrows. Dashed lines encircle cancer metastases. T = tumor; L = lung or liver. H&E scale bar = 100 µm.

Because CAFs are involved in cancer invasion and metastasis,^[^ ^28^, ^33^, ^39^, ^43^ ^]^ we studied the impact of Bmal1 deletion in cancer hosts on cancer metastasis. In the CRC model, genetic deletion of Bmal1 in mice markedly promoted pulmonary and liver metastasis (**Figure** **6**A–C). Approximately 83% of CRC tumor‐bearing Bmal1^−/−^ mice showed visible surface metastatic nodules, and only 33% of the control tumor–bearing Bmal1^+/+^ mice had visible metastases (Figure 6A). In addition to the increase of metastatic incidence, the total number of metastatic nodules on the surface of lungs and livers were also markedly increased (Figure 6B,C). Histological examination of lung and liver tissues further corroborated the presence of high numbers of microscopic metastases in CRC tumor‐bearing Bmal1^−/−^ mice versus the control tumor–bearing Bmal1^+/+^ mice (Figure 6B,C).

These findings were further validated in the PDAC cancer model in which pulmonary and hepatic metastases were markedly increased in tumor‐bearing Bmal1^−/−^ mice (Figure 6D–F). To further strengthen our conclusions, we isolated CAFs from the subcutaneous xenograft CRC and PDAC tumors by FACS‐sorting FAP^+^ cell populations. Co‐implantation of cancer cells and CAFs at the ratio of 1:1 resulted in marked increases of metastatic incidences in both Bmal1^+/+^ and Bmal1^−/−^ mice (Figure 6G–L). In PDAC tumor, co–implantation of Bmal1^−/−^CAFs and cancer cells resulted in metastatic development in nearly 90% of tumor‐bearing mice.

Immunohistochemical analysis of metastatic nodules in both lung and liver tissues from CRC and PDAC tumor‐bearing mice showed that metastases in Bmal1^−/−^ mice were enriched in stromal fibroblasts that were positive for FAP, *α*‐SMA, DESMIN, PDGFR*α*, and PDGFR*β* (Figure S7A–H, Supporting Information). In the cancer cell‐CAF‐co‐implantation model, isolated CAFs from the Bmal1^−/−^ mouse background exhibited a myofibroblast phenotype that boosted cancer metastasis (Figure S7I–N, Supporting Information). Similarly, the FAP^+^, *α*‐SMA^+^, DESMIN^+^, PDGFR*α* ^+^, and PDGFR*β* ^+^ fibrotic components in these metastases were also markedly increased (Figure S7I–N, Supporting Information). These data are also consistent with that CAFs being the primary drivers in TME for promoting cancer metastasis.

Cancer metastasis can occur at the very early stage of primary cancer development.^[^ ^53^ ^]^ In order to study the role of Bmal1^−/−^ CAFs during the early stage of metastasis, we employed a zebrafish metastasis model that allowed us to visualize cancer cell metastasis when the primary tumors were tiny.^[^ ^37^ ^]^ Relative to controls, co‐implantation of CRC cancer cells plus Bmal1^−/−^ CAFs markedly promoted cancer cell metastasis after 48 h (Figure S8A–F, Supporting Information). Nearly identical data were produced in a zebrafish PDAC metastasis model (Figure S8G–L, Supporting Information). Intriguingly, most metastatic CRC cancer cells were associated with Bmal1^−/−^ CAFs (Figure S8F, Supporting Information), demonstrating metastatic clusters of cancer cells and CAFs during the initial process of cancer metastasis.

### 2.8 Inhibition of the TGF‐*β* Signaling Blocks Cancer Metastasis

**Figure 7:**
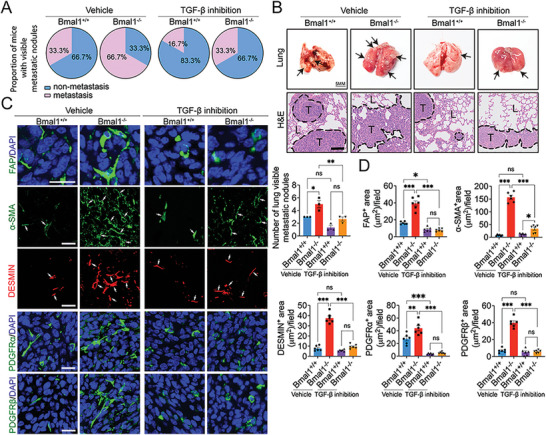
Anti‐metastatic effects of TGF‐*β* inhibitors in Bmal1^−/−^ mice. A) Quantification of visible metastases on the surface of lungs in vehicle‐ and SB‐431542‐treated tumors grown in Bmal1^+/+^ and Bmal1^−/−^ mice (*n* = 6 mice per group). B) Gross examination and H&E staining of lungs in vehicle‐ and SB‐431542‐treated CRC tumors grown in Bmal1^+/+^ and Bmal1^−/−^ mice. Apparent CRC metastatic nodules are indicated by arrows (scale bar = 5 mm). Quantification of visible metastatic nodules by H&E staining (*n* = 3 random fields per group). Dashed lines encircle cancer metastases. T = tumor; L = lung (scale bar = 100 µm). C) Representative micrographs of immunohistochemical staining of lung metastasis nodules of vehicle‐ and SB‐431542‐treated tumor in Bmal1^+/+^ and Bmal1^−/−^ mice with fibroblast markers: FAP (green); *α*‐SMA (green) plus CD31 (red); DESMIN (red) plus ENDOMUCIN (green); PDGFR*α* (green); and PDGFR*β* (green). D) Quantification of FAP^+^, *α*‐SMA^+^, DESMIN^+^, PDGFR*α* ^+^, and PDGFR*β* ^+^ signals (*n* = 6 random fields per group). Data presented as mean ± S.E.M. *P < 0.05; **P < 0.01; ***P < 0.001, ns = not significant; two‐tailed student *t*‐test and one‐way ANOVA. Scale bar = 50 µm.

To study the role of the TGF‐*β* signaling in cancer metastasis, we investigated TGF‐*β*R1 inhibition in our spontaneous CRC metastasis model. In vehicle‐treated control CRC tumor–bearing mice, high numbers of metastatic nodules were detected in Bmal1^−/−^ mice (**Figure** **7**A,B). However, treatment with SB‐431542 markedly decreased lung metastasis in tumor‐bearing in Bmal1^−/−^ mice (Figure 7A,B). The percentage of animals with pulmonary metastases in TGF‐*β*R1 inhibitor‐treated tumor‐bearing in Bmal1^−/−^ mice was identical to those in tumor‐bearing Bmal1^+/+^ mice (Figure 7A), consistent with TGF‐*β* signaling being responsible for the increased metastasis in CRC tumor–bearing Bmal1^−/−^ mice. Histological examination of lung tissues from tumor‐bearing mice corroborated the high numbers of metastatic lesions in Bmal1^−/−^ mice (Figure 7B). In response to TGF‐*β*R1 inhibition, pulmonary surface metastases were markedly decreased in Bmal1^−/−^ mice (Figure 7B). Immunohistochemistry analysis of metastatic nodules demonstrated marked mitigation of the FAP^+^, *α*‐SMA^+^, DESMIN^+^, PDGFR*α* ^+^, and PDGFR*β* ^+^ fibrotic components in TGF‐*β*R1‐treated Bmal1^−/−^ tumor‐bearing mice (Figure 7C,D). Together, these data demonstrate that the PAI‐1‐tPA/uPA‐plasmin‐TGF‐*β* signaling axis plays a central role in circadian rhythm–governed tumor fibrosis and cancer metastasis.

## 3 Discussion

Upon diagnosis of a cancerous disease, almost all patients encounter psychological trauma that triggers a series of physiologically dysfunctional responses, including fearfulness, eating disorders, and sleeping disorders. These disorders would result in circadian rhythmic disruption.^[^ ^13^ ^]^ While most cancer research has been focused on tumor growth, metastasis, and drug development, little is known about the effects of disruption of circadian rhythms in cancer progression, particularly metastasis. In this study, we have used a well‐accepted Bmal1^−/−^ mouse model^[^ ^54^ ^]^ of circadian disruption in the cancer hosts to study the role of clock rhythm in tumor growth, invasion, and metastasis. However, this conventional Bmal1^−/−^ mouse is a mixture of ON/OFF target effects with respect to the circadian clock. Recently, conditional Bmal1 knockout mice that lacked the BMAL1 protein during adult life experience different phenotypes relative to those of conventional Bmal1 knockout mice.^[^ ^55^ ^]^ An unexpected signaling pathway of the Bmal1KO‐PAI‐1‐tPA/uPA‐plasmin‐TGF‐*β*‐fibrosis axis has been discovered as a key regulatory loop in controlling tumor growth and metastasis. Consequently, targeting the TGF‐*β* signaling markedly inhibits tumor growth and metastasis. Thus, our work provides novel mechanistic insights into the circadian clock–governed TME in tumor growth and metastasis. Although genetic deletion of BMAL1 causes circadian rhythmic disruption, we cannot exclude non‐clock functions of Bmal1 in participating in cancer metastasis because genetic deletion of the core clock gene would have broad impacts on alterations of gene expression profiling in cancer hosts.

Several published works showed that BMAL1 protein expressed in cancer cells is positively correlated with invasion and metastasis.^[^ ^16^, ^18^ ^]^ In this regard, cancer cell–expressed BMAL1 acts as cancer invasion promoting protein. Perhaps, cancer cells utilize BMAL1‐targeted gene products to support their growth, invasion, and metastasis. Thus, BMAL1 serves as a core component of the master transcriptional machineries to control cancer cell motility and metastasis. This metastasis‐promoting effect of cancer cell–derived Bmal1 opposes the host stromal cell–expressed Bmal1‐regulated cancer metastasis. In fact, these opposing effects of Bmal1 in cancer cells and in host stromal cells are functionally in harmonized concordance in cancer metastasis. Malignant cells may not be dependent on the circadian rhythm as the relatively healthy stromal cells in TME. Their proliferation, migration, and invasion are regulated by genetic mutations and aberrant expressions of signaling molecules. By contrast, genetically stable stromal cells in cancer host are in part dependent on circadian rhythmicity to regulate their growth and functions. Thus, genetic disruption BMAL1 would produce a polarized effect on cancer cells and host stromal cells such as CAFs as described in our study. These polarized cellular behaviors between cancer cells and host cells further exacerbate cancer invasion and metastasis.

Bmal1 transcriptionally targets a myriad of genes and the Serpine1 (PAI‐1) promoter contains Bmal1‐binding E‐boxes.^[^ ^50^ ^]^ In the human PAI‐1 promoter–transfected endothelial cells, both CLOCK:BMAL1 and CLOCK:BMAL2 heterodimers activate the PAI‐1 promoter through E‐boxes.^[^ ^50^ ^]^ Under physiological conditions, Bmal1 upregulates PAI1 expression to inhibit plasmin activation by inhibiting tPA and uPA activity. Maintenance of plasmin homeostasis is crucial to control physiological functions of various cellular and molecular signaling components including prevention of excessive angiogenic sprouting, vascular leakiness, extravasation of inflammatory and immune cells, release of mobilized growth factors and cytokines, activation of certain cytokines, and thrombolysis.^[^ ^56^ ^]^ These plasmin‐regulated processes concomitantly occur in TME to restrict tumor growth and invasion.^[^ ^57^ ^]^ We show here that disruption of the circadian clock tips the balance of the cellular composition within TME toward an invasive phenotype by activation of plasmin. Although plasmin activates a cascade of responses in tumors, we have found that stimulation of tumor fibrosis is the prominent process in Bmal1^−/−^ mice. At the time of this writing, it is still unclear why fibrosis is the key phenotypic consequence of plasmin activation. However, it is clear that conversion of the inactive latent TGF‐*β* into active TGF‐ *β* by plasmin is responsible for the augmented tumor fibrosis. TGF‐ *β* potently stimulates tumor fibrosis and excessive fibrotic components increase the entire tumor volume, which manifests as an accelerated tumor growth rate. We provide experimental evidence that excessive fibrosis is responsible for the expansion of tumor volumes in Bmal1^−/−^ mice. It is interesting to mention that Bmal1 disruption–associated oxidative stress may link to the TGF‐*β* signaling. For example, an increased ROS level can activate TGF*β* signaling, which promotes cancer metastasis.^[^ ^58^ ^]^

TGF‐*β* potently instigates the transition of CAFs into a pro‐invasive phenotype of myoCAFs in tumors and myoCAFs facilitate tumor invasion and metastasis. TGF‐*β* has dual tumor‐promoting and inhibitory effects on cancer cells, depending on the tumor types. In our study, we primarily focus on the role of TGF‐*β* in cell migration, proliferation, and phenotypical changes of stromal fibroblasts. Published work from our lab and others demonstrates that myoCAFs can hijack cancer cells for intravasation and metastasis even in tumors that are not intrinsically metastatic.^[^ ^28^, ^43^, ^59^ ^]^ Thus, the composition and stromal cellular phenotypes in TME are key determinants for facilitating the initial intravasation step of cancer metastasis. Moreover, interactions between various cellular components, including inflammatory cells, vascular cells, fibrotic cells, and cancer‐associated adipocytes often coordinately synergize to promote metastasis. For example, plasmin activation potentially degrades the basement membrane of tumor microvessels and releases mobilized factors to promote motility of stromal cells and cancer cells. Remodeling microvessels by proteolytic degradation permits intravasation of tumor cells alone or in clusters with other stromal cells into the circulation. Metastatic cancer cells and CAFs form clusters in the circulation and these mosaic cell clusters co‐metastasize to distal tissues and organs.^[^ ^28^, ^43^, ^59^ ^]^ On the basis of these findings, it has been suggested that metastatic cancer cells as seeds carry their own fibrotic soil with them.^[^ ^59^ ^]^ Although the function of the circulating CAFs (cCAFs) in promoting cancer cell extravasation has not been investigated, it is reasonable to speculate that cCAFs contribute to extravasation of circulating tumor cells.

In addition to the stimulation of tumor fibrosis, deficiency of Bmal1 and its downstream circadian genes in mice increases fibrotic components in other tissues and organs, including lung, liver, and kidney,^[^ ^60^, ^61^, ^62^ ^]^ which may accommodate metastatic cancer cells. Enhanced fibrosis in these organs raises the possibility of preparation of metastatic niche formation since fibroblasts could serve as feeder cells to support metastatic tumor growth. If so, profound impacts of circadian disruption on cancer patients befall at several levels, including 1) promoting primary tumor growth; 2) facilitating tumor invasion; and 3) systemic preparation of metastatic niche formation. Thus, normalization of circadian rhythm oscillation provides an important approach for treating various types of cancers. Along this line of thinking, inhibition of tumor growth and metastasis by normalizing circadian rhythm would prolong timelines for cancer patients to receive conventional therapeutics such as chemotherapy, surgical operation, and radiation therapy, and other therapies, including immunotherapy, antiangiogenic therapy, and targeted therapies. In particular, combination therapies composed of clock normalization agents and other anticancer modalities may produce either additive or synergistic effects. Perhaps, targeting plasmin activation and TGF‐*β* drugs should also be considered in combination with other therapeutic modalities in combination therapeutic settings.

Together, we provide compelling evidence to demonstrate the essential role of the circadian clock in controlling tumor growth, and invasion and disruption of the circadian rhythm may have fatal consequences in promoting cancer metastasis. On the basis of our findings, we propose a new therapeutic paradigm of normalizing of the circadian clock either alone or in combination with other anticancer therapeutics, which might produce synergistic efficacy for treatment of various cancers.

## 4 Experimental Section

### Cell Culture

Murine MC38 colon cancer cell line was kindly provided by Dr. Rubén Hemández at the Gene Therapy Unit, Center for Applied Medical Research, University of Navarra, Pamplona, Navarra, Spain. Murine Panc02 PDAC cell line was kindly provided by Dr. Maximilian Schnurr from the Munich University, Germany. Above tumor cell lines were stably transfected with enhanced green fluorescent protein. Murine Hepa1‐6 hepatoma cell line was purchased from the ATCC (Manassas, VA, USA). Primary FAP^+^ CAFs were isolated by sorting cells from mouse MC38 and Panc02 tumor tissues using flow cytometry (FACS). MC38, Panc02, and Hepa1‐6 cells were cultured in Dulbecco's modified Eagle's medium (DMEM) (Cat. D6429, Sigma‐Aldrich) supplemented with 10% FBS, 100 U mL^−1^ penicillin, and 100 µg mL^−1^ streptomycin (Cat. P4333, Sigma‐Aldrich). FACS sorted primary FAP^+^ CAFs were cultured in DMEM/F12 (Cat. 11320‐074, Gibco) with 10% FBS, 100 U mL^−1^ penicillin, and 100 µg mL^−1^ streptomycin.

### Mouse Tumor Models and Drug Treatment

Bmal1^+/−^ mice^[^ ^63^ ^]^ as breeding founders were kindly provided by Dr. Garret A. FitzGerald at the Institute for Translational Medicine and Therapeutics, Perelman School of Medicine, University of Pennsylvania, USA. Bmal1^−/−^ and Bmal1^+/+^ mice were confirmed by genotyping of ear tissue. Genotyping was performed by a PCR‐based method using primers OL2646: 5′‐CCACCAAFCCCAFCAACRCA‐3′; OL2657: 5′‐ATTCGGCCCCCTATCTTCTGC‐3′; and OL278: 5′‐TCGCCTTCTATCGCCTTCTTCTTGACG‐3′. C57BL/6 wild type mice were purchased from the Janvier Laboratory, Germany. Mice were housed in animal facilities with constant temperature (20 ± 2 °C), humidity (50 ± 10%), and a 12 h‐light and 12 h‐dark exposure cycle each day. Approximately 3 × 10^6^ tumor cells in 100 µL Phosphate‐buffered saline (PBS) were subcutaneously implanted into the dorsal region of each Bmal1^−/−^ and Bmal1^+/+^ mouse. Tumor volumes were measured with a caliper and calculated according to the standard formular (length × width^2^ × 0.52). When tumor volumes reached about 2.0 cm^3^, mice were euthanized by inhalation of overdose of isoflurane (Attane vet, Cat. QN01AB06, VM Pharma) and tumor tissues were dissected. For the orthotopical tumor model by implanting tumor cells into livers, a right subcostal incision was created and ≈1 × 10^6^ Hepa1‐6 tumor cells in PBS were injected to the left lobe of the liver in each Bmal1^−/−^ and Bmal1^+/+^ mouse. After operation, the pain killer was delivered in the animals. Three weeks after tumor implantation, tissues and organs were harvested. For TGF‐*β* inhibition experiment, SB431542, a selective activin receptor‐like kinase 5 inhibitor (Cat. S1067, Selleck Chemicals), at the dose of 10 mg kg^−1^ was intraperitoneally injected three times per week into mice. Dimethyl sulfoxide (Cat.276855, Sigma‐Aldrich) was used as a vehicle control. Treatment was initiated when tumor volumes reached to 0.15–0.20 cm^3^ and terminated when tumors reached to 2.0 cm^3^. At the end of each experiment, fresh tumor tissues were collected for further analysis. For histological studies, the samples were fixed with 4% paraformaldehyde.

Approximately 3 × 10^6^ tumor cells in 100 µL PBS were subcutaneously implanted into each Bmal1^−/−^ and Bmal1^+/+^ mouse. When tumor volumes reached about 2.0 cm^3^, tumor tissues were surgically removed. Pain killer was delivered to each mouse after operation. Six weeks after tumor removal, mice were euthanized and organs were dissected for detection of visible metastases, which were subsequently validated and examined by histology and immunohistochemistry. For co‐implantation of tumor cells and or plus CAFs, ≈2 × 10^6^ tumor cells and 2 × 10^6^ FAP^+^CAFs were subcutaneously co‐injected into wild type C57BL/6 mice. When tumor volumes reached 2.0 cm^3^, tumor tissues were surgically removed. Pain killer was delivered to each mouse after operation. Six weeks after tumor removal, organs were dissected for detection of visible metastases, which were subsequently validated and examined by histology and immunohistochemistry.

### Metastatic Model in Zebrafish

All zebrafish work was performed at the Zebrafish Core Facility of the Karolinska Institutet and experiments were conducted under the regulation of the facility. Wild‐type AB strains of zebrafish were raised at 28 °C under standard conditions. At 24 h‐post‐fertilization (hpf), zebrafish embryos were transferred to an E3 medium containing 0.2 mmol L^−1^ 1‐phenyl‐2‐thiourea (Cat. 189 235, Sigma‐Aldrich) to prevent pigmentation. Embryos were anesthetized with 0.04 mg mL^−1^ of tricaine (Cat. MS‐222, Sigma‐Aldrich) at 48 hpf prior to microinjection. CAFs isolated from Bmal1^−/−^ and Bmal1^+/+^ mice were labeled with 1 µg mL^−1^ 1′‐dioctadecyl‐3, 3, 3′, 3′‐tetramethylindocarbocyanine per‐chlorate (DiI, Cat. 42 364, Merck) in vitro. Approximately 100–200 CAFs and 100–200 GFP tumor cells at the ratio of 1:1 were subsequently injected into the perivitelline space of each eligible embryo. Zebrafish embryos were transferred into 24‐well plates (1 embryo per well) and incubated at 33 °C for additional 48 h. Micrographic images were taken at 48 h after cell injection. Positive signals were detected by using a fluorescence microscope equipped with a camera (Nikon, DS‐QilMC). Images were analyzed by using an Adobe Photoshop software (CS6, Adobe) program.

### Whole‐Mount Staining

Tumor tissue samples were cut into thin pieces and digested with 20 mm proteinase K for 5 min, followed by incubation for 30 min with 100% methanol. Tissue samples were further incubated at 4 °C overnight with 0.3% Triton X‐100 PBS containing 3% skim milk. Samples were rigorously washed with PBS and subsequently incubated overnight with a combination of a rat anti‐mouse CD31 (1:200, Cat. 553 370, BD Pharmingen) antibody and a rabbit polyclonal anti‐NG2 (1:200, Cat. AB5320, Merck) antibody. After rigorous washes with PBS, species‐matched secondary antibodies were added for incubation overnight at 4 °C. After washing with PBS, stained tissue samples were mounted by a vectashield‐mounting medium (H100, Vector Laboratories). Images were taken by confocal microscopy (Nikon, DS‐QilMC). 3D images of tumor vessels were analyzed. Positive signals of CD31 and NG2 were calculated by Adobe Photoshop software.

### Immunohistochemistry

Paraffin‐embedded tumor tissues were cut into 5 µm thick sections. Tissue slides were de‐paraffinized by Tissue‐Clear (Cat. 1466, Sakura) and rehydrated with sequential steps in 98‐95‐70% ethanol. After boiling with antigen unmasking solution (H3300, Vector) for 20 min and subsequently blocked with 4% serum, tissue slides were stained at 4 °C overnight with a rat anti‐mouse FAP (1:200, Cat. MAB9727, R&D system) antibody, a mouse anti‐mouse *α*‐SMA (1:200, Cat. MA5‐11547, Thermo Fisher Scientific) antibody, a goat anti CD31 (1:400, Cat. AF3628, R&D system) antibody, a rat anti ENDOMUCIN (1:400, Cat. 14 585 185, eBioscience) antibody, a rabbit anti DESMIN (1:200, Cat. 0 4585, Millipore) antibody, a goat anti PDGFR*α* (1:200, Cat. AF1062, R&D system) antibody, a rabbit anti PDGFR*β* (1:200, Cat. 13449‐1‐AP, Proteintech) antibody, a rabbit CAIX (1:200, Cat. NB100‐417, NOVUS) antibody, a rat anti Ki67 (1:400, Cat. 14‐5698‐82, eBioscience) antibody, a rabbit anti F4/80 (1:200, D2S9R, Cell Signaling TECHNOLOGY) antibody, a mouse anti CD80 (1:200, Cat. NBP2‐25255SS, NOVUS BIOLOGICALS) antibody, and a mouse anti CD206 (1:200, Cat. 60143‐1‐Ig, Proteintech) antibody, followed by staining for 1 h at room temperature with species‐matched secondary antibodies. Apoptotic staining was performed according to the manufacturer's protocol (Cat. 11 684 795 910, Roche). Nuclei were counterstained with DAPI (Cat. D21490, Invitrogen). Slides were mounted by vectashield mounting medium. Positive signals were detected by fluorescence microscope (Nikon, DS‐QilMC) equipped with a camera. Images were analyzed by Adobe Photoshop software.

### H&E Staining

The paraffin‐embedded tissues were cut, deparaffinized, and rehydrated as described. Tissue slides were stained with hematoxylin (6 765 009; Thermo Fisher Scientific), followed by eosin (HT110116; Sigma‐Aldrich). After being dehydrated with 95–99% ethanol, slides were mounted with PERTEX (00801, HistoLab). Stained tissues were photographed using a light microscope (Nikon Eclipse TS100) equipped with a camera (DS‐Fi1; Nikon) and software (NIS‐Elements F3.0).

### Enzyme‐Linked Immunosorbent Assay

Enzyme‐linked immunosorbent assay (ELISA) was used for measuring mouse active PAI‐1 and active TGF‐*β* proteins. To detect active PAI‐1protein levels, the whole tumor tissue, SVF, CAFs supernatant, and tumor‐bearing mice plasma were analyzed according to the manufacturer's protocol (Cat. MBS135529, MyBioSource). To detect active TGF‐*β* protein level, the whole tumor tissue and tumor‐bearing mice plasma were analyzed according to the manufacturer's protocol (Cat. 437 707, Biolegend). Protein level was justified with the standard curve. Absorbance values were detected at 450 nm using a microplate reader and values were calculated by using the formula obtained from the trendline.

### Immunoblotting

Total proteins from SVF, whole tumor tissue, and CAFs were extracted using RIPA lysis buffer (Cat. R0278, Sigma‐Aldrich) and an extraction buffer (Cat. 89 900, Thermo Fisher Scientific), containing a Protease Inhibitor Cocktail (Cat. 87 785, Thermo Fisher Scientific) and a Phosphorate Inhibitor Cocktail (Cat. 1 862 495, Thermo Scientific). An equal amount of protein from each sample was loaded onto Mini‐PROTEAN TGX Gels (Cat. 4 561 086, BIO‐RAD), following by transferring onto PVDF membranes (Cat. 1 620 184, BIO‐RAD). Immunoblotting was carried out with primary antibodies specific for PAI‐1 (Cat. Ab66705, Abcam), *α*‐SMA (Cat. MA5‐11547, Thermo Fisher Scientific), FAP (Cat. NBP2‐89135, Novus Biologicals), PDGFR*β* (Cat. 13449‐1‐AP, proteintech), uPA (Cat. 10147‐1‐AP, Proteintech), tPA (Cat. PA5‐85186, Invitrogen), and *β*‐actin (Cat. 612 657, BD Biosciences). The primary antibody against *β*‐actin was used to adjust the sample loading. The secondary antibodies included a donkey anti‐mouse antibody conjugated with IRDye 680RD (926‐68072, LI‐COR Biosciences) and a donkey anti‐rabbit antibody conjugated with IRDye 800CW (926‐32213, LI‐COR Biosciences). Densitometry analysis was performed using the Odyssey CLX Imaging System (LI‐COR Biosciences).

### uPA Activity Assay

To detect uPA activity in CRC tumor, SVF, CAF supernatant, and CAF cells, the samples were performed according to the manufacturer's protocol (uPA, Cat. MAK185, Sigma‐Aldrich) with standard curve. Fluorescence intensities were measured under ex = 350/em = 450 nm with multiple timepoints by a microplate reader (Victor X3, PerkinElmer) and values were calculated by using the formula obtained from the trendline.

### tPA Activity Assay

To detect tPA activity in CRC tumor, SVF, CAF supernatant, and CAF cells, the samples were performed according to the manufacturer's protocol (tPA, Cat. K178, BioVision) with standard curve. Absorbance values were detected at 450 nm using a microplate reader (FLUOstar Omega, BMG LABTECH) and values were calculated by using the formula obtained from the trendline.

### Plasmin Activity Assay

To detect plasmin activity in CRC tumor and tumor‐bearing mice plasma, the samples were performed according to the manufacturer's protocol (Cat. MAK244, Sigma‐Aldrich) with the standard curve. Absorbance values were detected at 450 nm using a microplate reader (FLUOstar Omega, BMG LABTECH) and values were calculated by using the formula obtained from the trendline.

### CAF and SVF Isolation

Fresh tumor tissues from Bmal1^+/+^ and Bmal1^−/−^ mice were cut into small pieces and incubated in a combination with 0.15% type I collagenase (Cat. C0130‐500MG, Sigma‐Aldrich) and 0.15% type II collagenases (Cat. C6885‐1G, Sigma‐Aldrich) in PBS at 37 °C for 1 h. Digested tissues were filtered with a 100 µm cell strainer and followed by a 70 µm cell strainer. For FAP^+^ CAFs sorting, after centrifugation, single‐cell suspensions from each sample were stained on ice for 15 min with a rat anti‐mouse FAP (1:50, Cat. MAB9727, R&D system) antibody, followed by an Alexa Fluor 647‐conjugated donkey anti‐Rat secondary antibody (Cat. A48272, Thermo Fisher Scientific). The stained samples were filtered with a 40 µm cell strainer. CAFs were sorted by FACS Aria III (BD Bioscience) using a BD FACSDiva software (BD Bioscience). The tumor cells labeled with GFP and non‐labeled cells were applied to exclude from sorting gate. For SVF sorting, single‐cell suspensions from each sample were sorted. Wild‐type and GFP tumor cells were applied to exclude from sorting gate.

### RNA Sequencing

For RNA sequencing, total RNAs were collected from isolated tumor SVF and were measured by NanoDrop One Microvolume UV–vis Spectrophotometer (Thermo Fisher SCIENTIFIC). The quality of RNA samples was assessed by agarose gel electrophoresis and Agilent 2100 Bioanalyzer (Agilent technologies). All samples displayed a 260/280 ratio around 2.0. cDNA libraries were constructed and sequenced by Sinotech Genomics Co., Ltd (Shanghai, China). Briefly, 200 ng RNA for each group was used for the library construction by a TruseqTM RNA sample prep Kit (Illumina, USA). The constructed DNA was enriched by PCR amplification, followed by purification with Certified Low Range Ultra Agarose (Bio‐Rad, USA) gel electrophoresis. Clusters were generated by cBot with the library diluted to 10 pm and were sequenced on the IlluminaNovaSeq 6000 (Illumina, USA). After sequencing, de novo assembly and sequence analyses were performed. The raw paired‐end reads were mapped to the reference genome (GRCm38.102) using Hierarchical Indexing for Spliced Alignment of Transcripts, version 2.0.5. The output SAM (sequencing alignment/map) files were converted to BAM (binary alignment/map) files and sorted using SAMtools (version 1.3.1). Gene abundance was expressed as fragments per kilobase of exon per million reads mapped. Stringtie software was used to count the fragments within each gene, and TMM algorithm was used for normalization. Differential expression analysis for mRNA was performed using R package edgeR. Differentially expressed RNAs with |log2(FC)| value >1 and *p* value <0.05, considered as significantly modulated, were retained for further analysis. The data was deposited in gene expression omnibut under accession code GSE217250.

### RNA Extraction and Quantitative Real‐Time PCR (qPCR)

Total RNAs were isolated from tumor tissues, FACS‐sorted SVF, and cultured cells for qPCR analysis. RNAs from each sample were reversely transcribed using a RevetAid H minus First Strand cDNA synthesis kit (Cat. K1632, Thermo Fisher SCIENTIFIC). Reverse transcription was performed at 42 °C for 60 min, followed by 70 °C for 5 min to inactivate the enzyme activity. Samples were stored at −20 °C and subsequently subjected to qPCR using an ABI Prism 7500 system (Applied Biosystems). Each sample in a triplicate was subjected to a 15 µL reaction containing SYBR Green (Cat. A25742, Thermo Fisher SCIENTIFIC), 150 nm forward and reverse primers, and 1 µL cDNA. The qPCR protocol was executed for 40 cycles and each cycle consisted of denaturation at 95 °C for 15 s, annealing at 60 °C for 1 min, and extension at 72 °C for 1 min. The primer pairs specific for various genes used were included in Table S1, Supporting Information.

### Cell Motility

Approximately 1 × 10^4^ per well of FAP^+^ CAFs isolated from tumor‐bearing Bmal1^+/+^ and Bmal1^−/−^ mice were seeded into a 6 cm dish. Three to four colonies per dish were observed, and images were recorded every 4 h for a consecutive 48 h. Cells and motility data obtained by manual tracking of cells (Manual Tracking, ImageJ).

### Statistical Analysis

Collected data analyses were performed using Excel (Microsoft 365, USA) and GraphPad Prism (GraphPad, USA). Data presented as mean determinants ± SEM. Statistical computations were performed using the standard two‐tailed Student *t*‐test and the one‐way ANOVA, *P < 0.05, **P < 0.01, and ***P < 0.001 were considered statistically significant. Statistics used in individual data were indicated in each figure.

### Study Approval

All animal studies were approved by the Northern Stockholm Ethical Committee for animal experiments in Sweden (6196‐2019) and the Ethical Committee for animal experiments of the Fudan University in China (20 190 430).

## Conflict of Interest

The authors declare no conflict of interest.

## Author Contributions

J.W. and X.J. contributed equally to this work. Y.C. generated initial idea. Y.C. designed the study and J.W. performed most experiments and data analyses. X.J., Q.D., X.S., and K.H. performed some experiments and analyses. J.G., X.H., and K.H. performed some analyses. C.Z., W.T., G.A.F., Y.Y., and L.D.J. significantly contributed to discussion, data analysis, and material supply. Y.C. wrote the manuscript and J.W. wrote the Materials and Methods.

## Supporting information

Supporting Information

## Data Availability

The data that support the findings of this study are available from the corresponding author upon reasonable request.
