# Supplementary material for: Disruption of the Clock Component Bmal1 in Mice Promotes Cancer Metastasis through the PAI‐1‐TGF‐β‐myoCAF‐Dependent Mechanism
Source: Adv Sci (Weinh). 2023 Jun 17;10(24):2301505. doi: 10.1002/advs.202301505 (PMC10460897; doi:10.1002/advs.202301505)
Supplement: Supplementary file 1 — Supporting Information [file ADVS-10-2301505-s001.pdf]

ADVANCED SCIENCE
Open Access

Supporting Information

for *Adv. Sci.*, DOI 10.1002/advs.202301505

Disruption of the Clock Component Bmal1 in Mice Promotes Cancer Metastasis through the PAI-1-TGF-β-myoCAF-Dependent Mechanism

*Jieyu Wu, Xu Jing, Qiqiao Du, Xiaoting Sun, Kristian Holgersson, Juan Gao, Xingkang He, Kayoko Hosaka, Chen Zhao, Wei Tao, Garret A. FitzGerald, Yunlong Yang, Lasse D. Jensen and Yihai Cao**

Supporting Information

Disruption of the clock component Bmal1 in mice promotes cancer metastasis through the PAI-1-TGF-β-myoCAF-dependent mechanism

*Jieyu Wu[#], Xu Jing[#], Qiqiao Du, Xiaoting Sun, Kristian Holgersson, Juan Gao, Xingkang He, Kayoko Hosaka, Chen Zhao, Wei Tao, Garret A. FitzGerald, Yunlong Yang, Lasse D. Jensen and Yihai Cao**

*Correspondence, galley proofs and reprint requests should be primarily addressed to: Yihai Cao, M.D., Ph.D., Department of Microbiology, Tumor and Cell Biology, Karolinska Institutet, 171 77 Stockholm, Sweden. Tel: (+46)-8-5248 7596, Fax: (+46)-8-33 13 99, E-mail: yihai.cao@ki.se

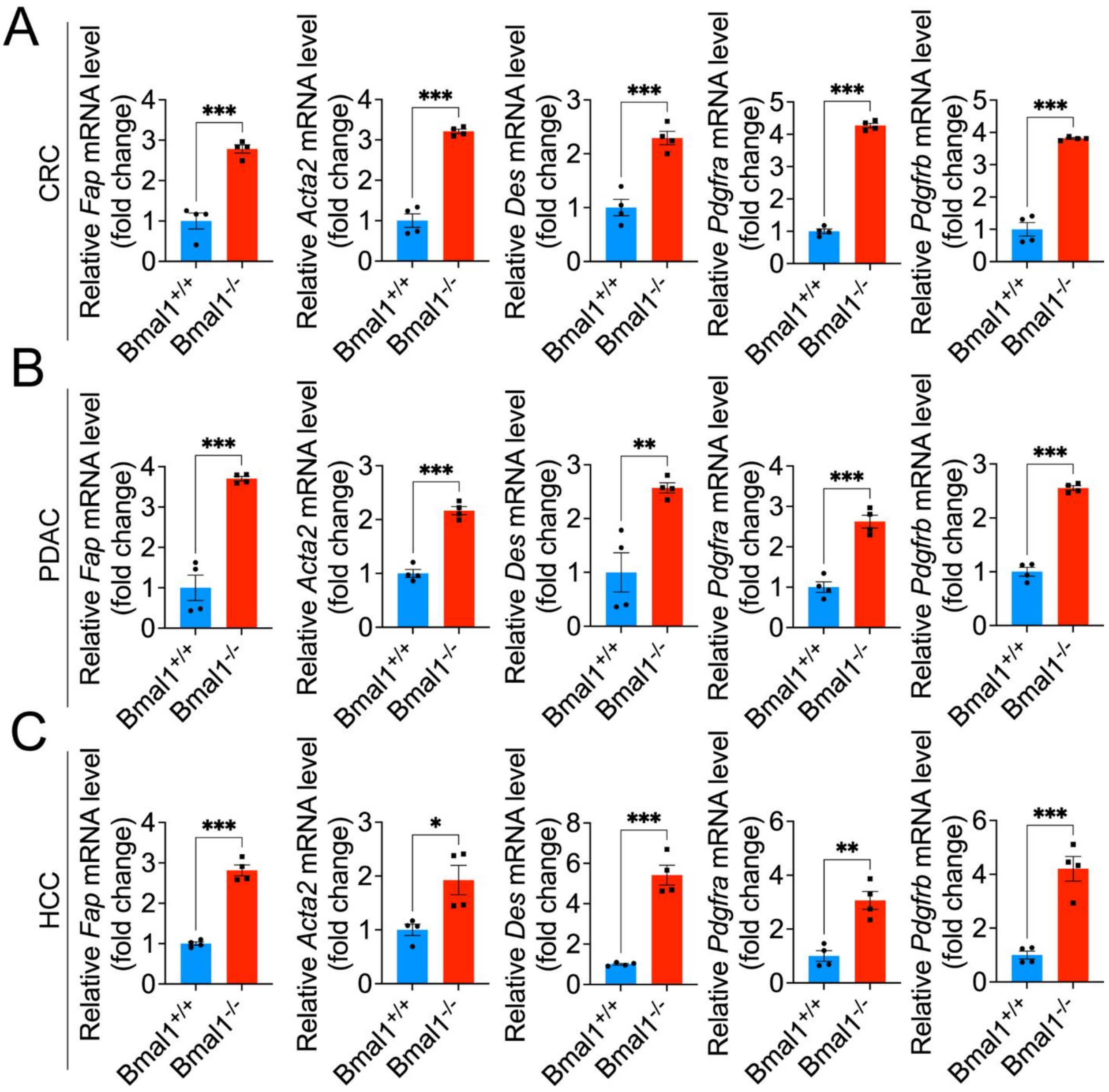

Supplemental Figure 1. mRNA expression levels of fibroblast-related markers in tumors.

A. qPCR quantification of mRNA levels of *Fap*, *Acta2*, *Des*, *Pdgfra* and *Pdgfrb* in CRC tumors in Bmal1$^{+/+}$ and Bmal1$^{-/-}$ mice (n = 4 samples per group).

B. qPCR quantification of mRNA levels of *Fap*, *Acta2*, *Des*, *Pdgfra* and *Pdgfrb* in PDAC tumors in Bmal1$^{+/+}$ and Bmal1$^{-/-}$ mice (n = 4 samples per group).

C. qPCR quantification of mRNA levels of *Fap*, *Acta2*, *Des*, *Pdgfra* and *Pdgfrb* in HCC

tumors in $Bmal1^{+/+}$ and $Bmal1^{-/-}$ mice (n = 4 samples per group).

Data presented as mean ± s.e.m. *$P < 0.05$; **$P < 0.01$; ***$P < 0.001$; two-tailed student *t*-test.

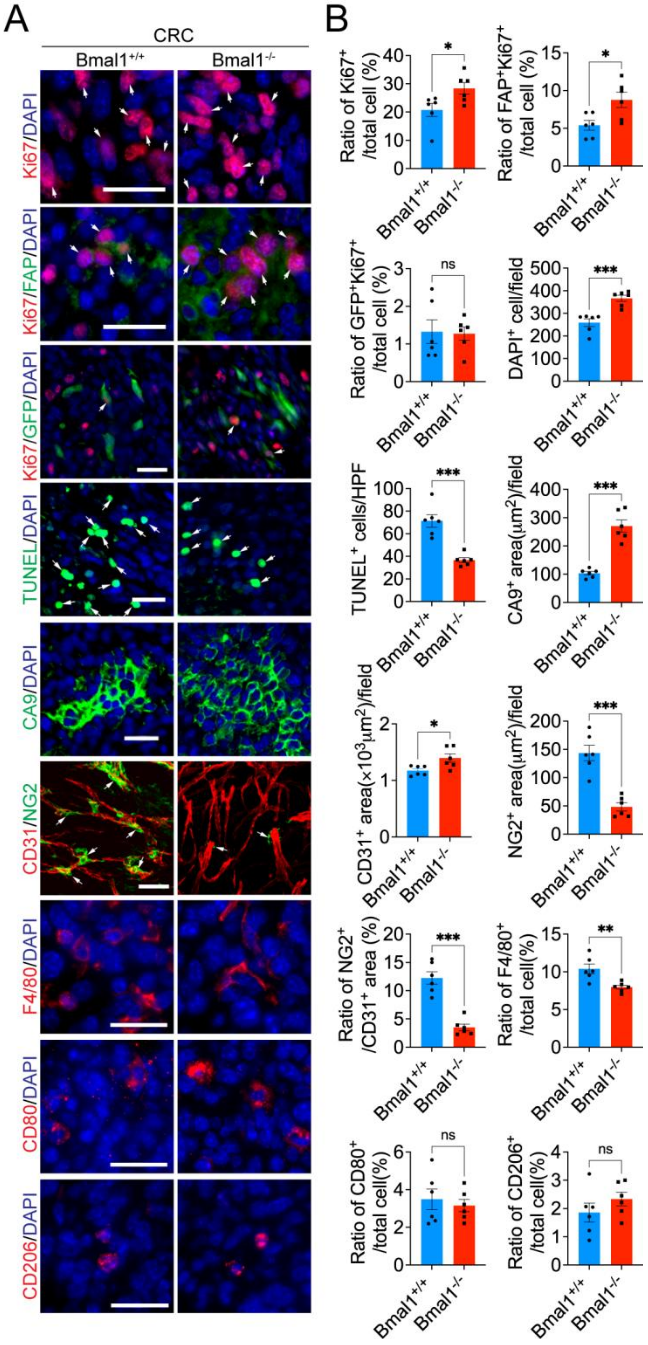

Supplemental Figure 2. Cell proliferation, cell death, hypoxia, microvessels, and inflammation of CRC tumors grown in Bmal1$^{+/+}$ and Bmal1$^{-/-}$ mice.

A. Representative micrographs of immunohistochemical staining of CRC tumor tissue stained with Ki-67 (red), FAP (green), CA9 (green), CD31 (red), NG2 (green), F4/80 (red), CD80

(red), CD206 (red), DAPI (blue), fluorochrome-based TUNEL assay (green) and green fluorescent protein (GFP) signal-labeled tumor cells (green).

B. Quantification of Ki-67$^+$, FAP$^+$ plus Ki-67$^+$, GFP$^+$ plus Ki-67$^+$, DAPI, TUNEL$^+$, CA9$^+$, CD31$^+$, NG2$^+$, NG2$^+$ plus CD31$^+$, F4/80$^+$, CD80$^+$, and CD206$^+$ in Bmal1$^{+/+}$ and Bmal1$^{-/-}$ mice (n = 6 random fields per group).

Data presented as mean determinants from random fields ± s.e.m. *$P < 0.05$; **$P < 0.01$; ***$P < 0.001$, ns = not significant; two-tailed student *t*-test. Scale bar = 50 µm.

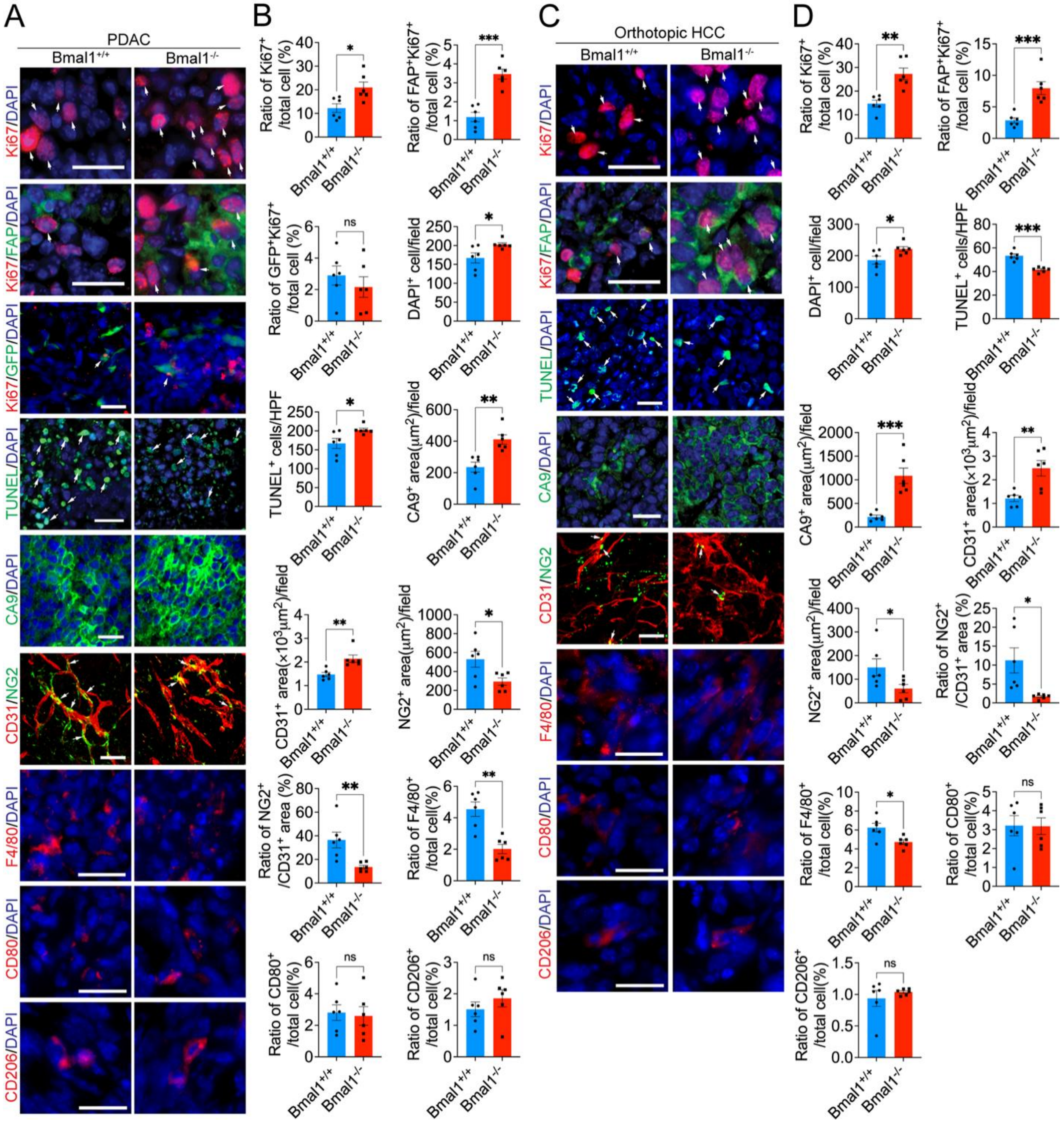

Supplemental Figure 3. Cell proliferation, cell death, hypoxia, microvessels, and inflammation of PDAC and HCC tumors grown in Bmal1$^{+/+}$ and Bmal1$^{-/-}$ mice.

A. Representative micrographs of immunohistochemical staining of PDAC tumor tissues from Bmal1$^{+/+}$ and Bmal1$^{-/-}$ mice stained with Ki-67 (red), FAP (green), fluorochrome-based

TUNEL assay (green), CA9 (green), CD31 (red), NG2 (green), F4/80 (red), CD80 (red), CD206 (red), DAPI (blue), and GFP signal-labeled tumor cells (green).

B. Quantification of Ki-67$^+$, FAP$^+$ plus Ki-67$^+$, GFP$^+$ plus Ki-67$^+$, DAPI$^+$, TUNEL$^+$, CA9$^+$, CD31$^+$, NG2$^+$, NG2$^+$ plus CD31$^+$, F4/80$^+$, CD80$^+$, and CD206$^+$ (n = 6 random fields per group).

C. Representative micrographs of immunohistochemical staining of orthotopic HCC tumor tissues stained with Ki-67 (red), FAP (green), CA9 (green), CD31 (red), NG2 (green), F4/80 (red), CD80 (red), CD206 (red), DAPI (blue) and fluorochrome-based TUNEL assay (green).

D. Quantification of Ki-67$^+$, FAP$^+$ plus Ki-67$^+$, DAPI, TUNEL$^+$, CA9$^+$, CD31$^+$, NG2$^+$, NG2$^+$ plus CD31$^+$, F4/80$^+$, CD80$^+$, and CD206$^+$ in Bmal1$^{+/+}$ and Bmal1$^{-/-}$ mice (n = 6 random fields per group).

Data presented as mean determinants from random fields ± s.e.m. *$P < 0.05$; **$P < 0.01$; ***$P < 0.001$, ns = not significant; two-tailed student *t*-test. Scale bar = 50 μm.

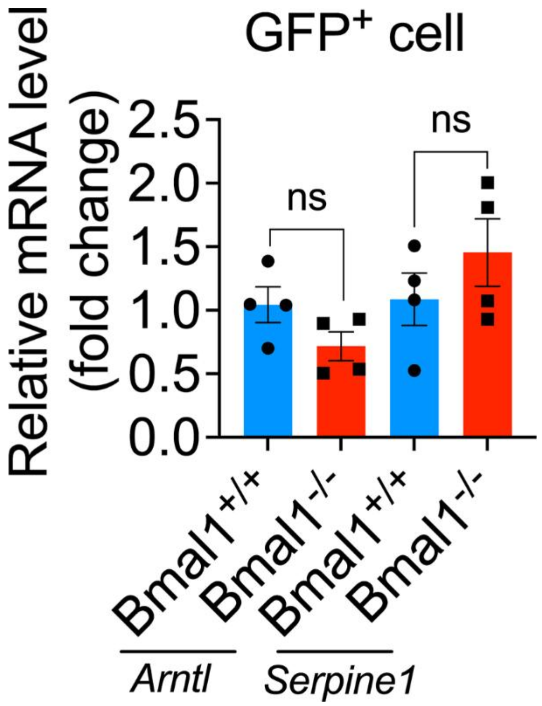

Supplemental Figure 4. mRNA expression levels of *Arntl* and *Serpine1* in FACS sorted GFP$^+$ cells from CRC tumor.

qPCR quantification of mRNA levels of *Arntl (Bmal1)* and *Serpine1* in FACS-sorted-GFP$^+$ tumor cells from CRC tumor in Bmal1$^{+/+}$ and Bmal1$^{-/-}$ mice (n = 4 samples per group).

Data presented as mean ± s.e.m. ns = not significant; two-tailed student *t*-test.

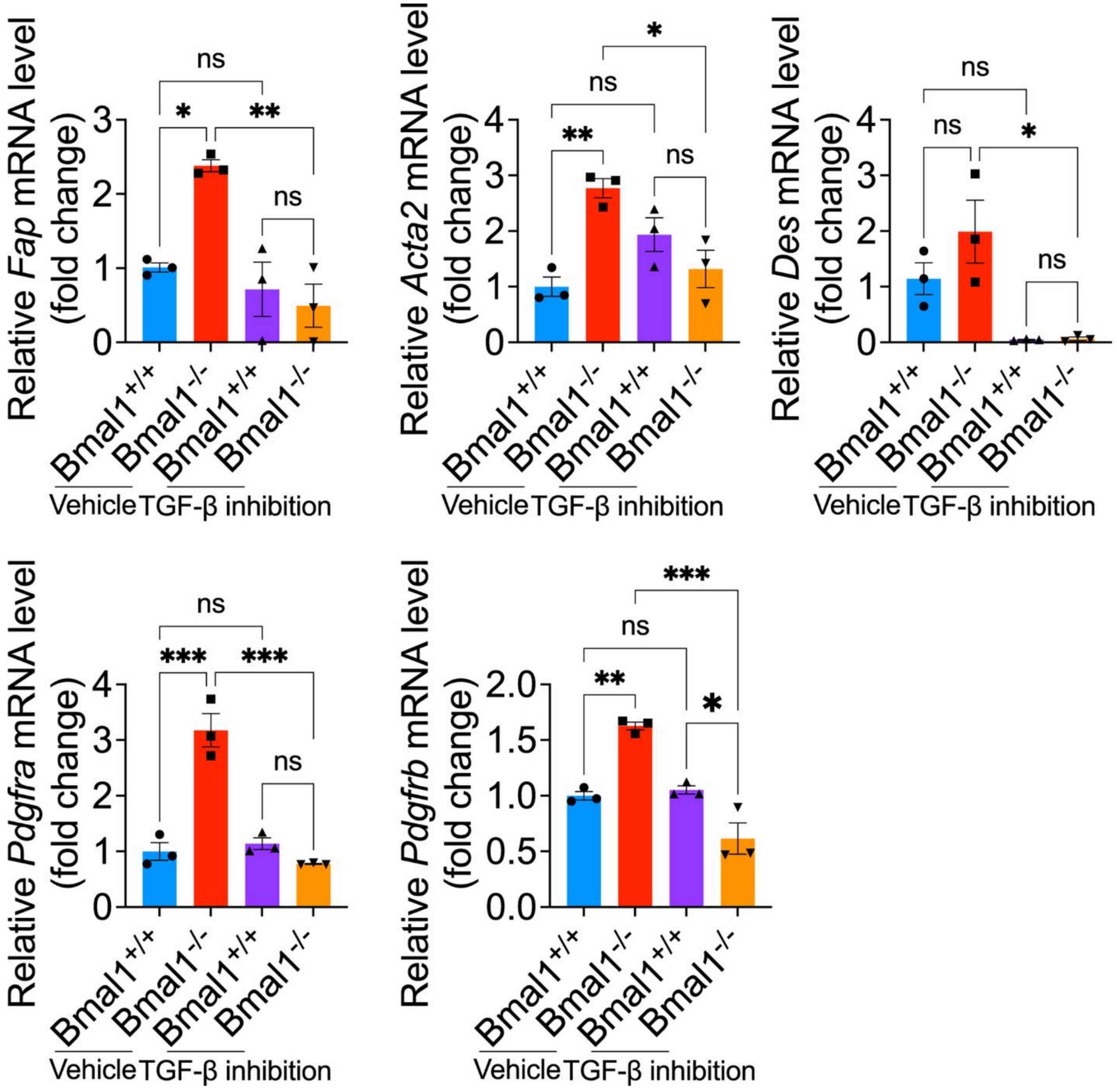

Supplemental Figure 5. mRNA expression levels of fibrotic markers in vehicle- and SB-431542-treated CRC tumors grown in Bmal1$^{+/+}$ and Bmal1$^{-/-}$ mice.

qPCR quantification of mRNA levels of *Fap*, *Acta2*, *Des*, *Pdgfra* and *Pdgfrb* in vehicle- and SB-431542-treated CRC tumors grown in Bmal1$^{+/+}$ and Bmal1$^{-/-}$ mice (n = 3 samples per group).

Data presented as mean ± s.e.m. $*P < 0.05$; $**P < 0.01$; $***P < 0.001$, ns = not significant; one-way ANOVA.

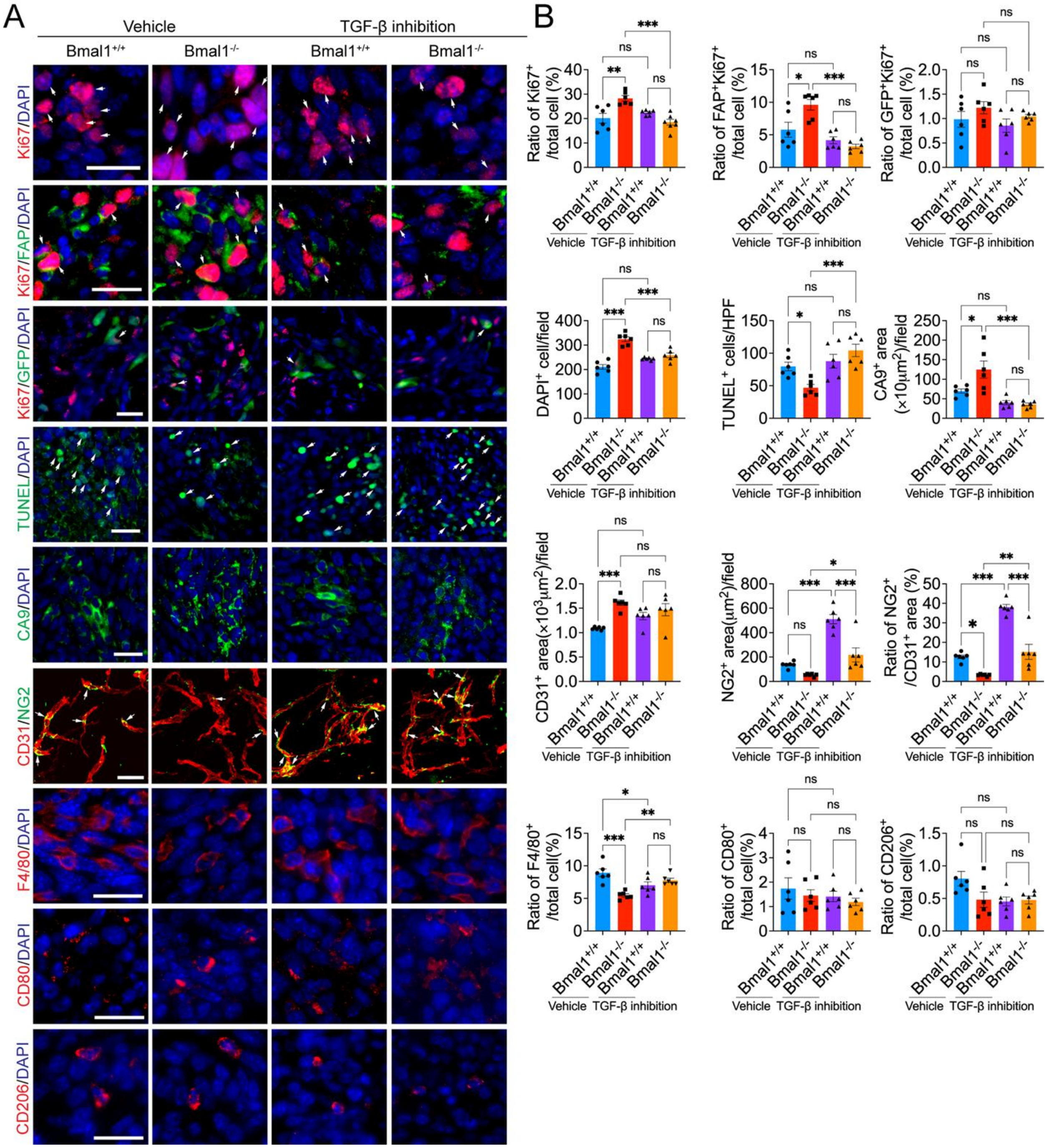

Supplemental Figure 6. Cell proliferation, cell death, hypoxia, microvessels, and inflammation of vehicle- and SB-431542-treated CRC tumors grown in Bmal1$^{+/+}$ and Bmal1$^{-/-}$ mice.

A. Representative micrographs of immunohistochemical staining of vehicle- and SB-431542-treated CRC tumor tissues grown in Bmal1$^{+/+}$ and Bmal1$^{-/-}$ mice stained with Ki-67 (red), FAP (green), fluorochrome-based TUNEL assay (green), CA9 (green), CD31 (red), NG2 (green), F4/80 (red), CD80 (red), CD206 (red), DAPI (blue), and GFP signal-labeled tumor cells (green).
B. Quantification of positive signals of vehicle- and SB-431542-treated CRC tumor tissues in Bmal1$^{+/+}$ and Bmal1$^{-/-}$ mice stained with Ki-67, FAP plus Ki-67, GFP plus Ki-67, DAPI, TUNEL, CA9, CD31, NG2, NG2 plus CD31, F4/80, CD80, and CD206 (n = 6 random fields per group).

Data presented as mean determinants from random fields ± s.e.m. *$P < 0.05$; **$P < 0.01$; ***$P < 0.001$, ns = not significant; one-way ANOVA. Scale bar = 50 μm.

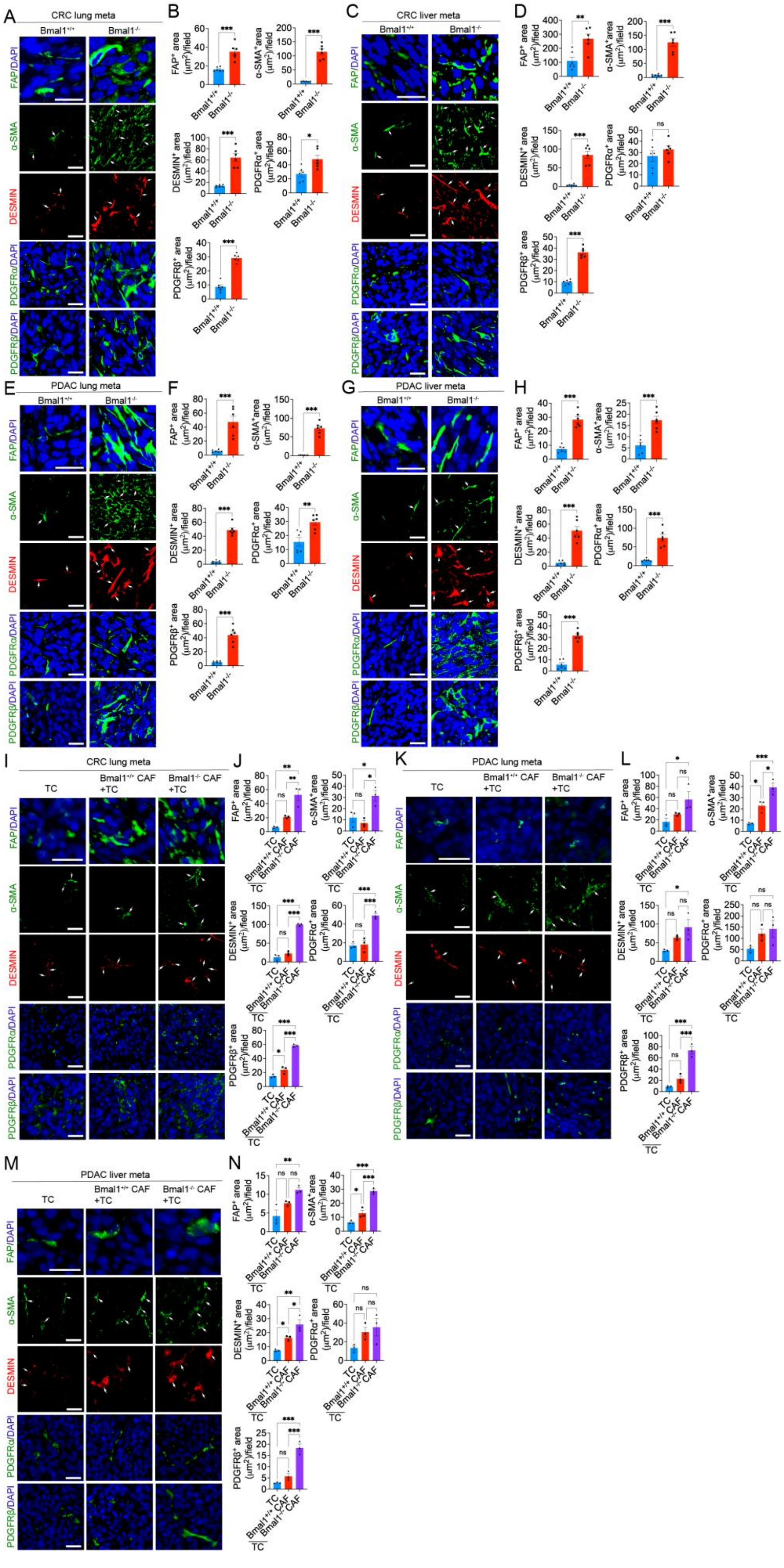

Supplementary Figure 7. Hyperfibrosis in metastatic nodules of $Bmal1^{-/-}$ mice.

A. Representative micrographs of immunohistochemical staining of lung metastatic lesions of CRC tumor in $Bmal1^{+/+}$ and $Bmal1^{-/-}$ mice with fibroblast markers: FAP (green); α-SMA (green); DESMIN (red); PDGFRα (green); and PDGFRβ (green).

B. Quantification of FAP^{+}, α-SMA^{+}, $DESMIN^{+}$, PDGFRα^{+} and PDGFRβ^{+} signals (n = 6 random fields per group).

C. Representative micrographs of immunohistochemical staining of liver metastatic lesions of CRC tumor in $Bmal1^{+/+}$ and $Bmal1^{-/-}$ mice with fibroblast markers: FAP (green); α-SMA (green); DESMIN (red); PDGFRα (green); and PDGFRβ (green).

D. Quantification of FAP^{+}, α-SMA^{+}, $DESMIN^{+}$, PDGFRα^{+} and PDGFRβ^{+} signals (n = 6 random fields per group).

E. Representative micrographs of immunohistochemical staining of lung metastatic lesions of PDAC tumor $Bmal1^{+/+}$ and $Bmal1^{-/-}$ mice with fibroblast markers: FAP (green); α-SMA (green) plus CD31 (red); DESMIN (red) plus ENDOMUCIN (green); PDGFRα (green); and PDGFRβ (green).

F. Quantification of FAP^{+}, α-SMA^{+}, $DESMIN^{+}$, PDGFRα^{+} and PDGFRβ^{+} signals (n = 6 random fields per group).

G. Representative micrographs of immunohistochemical staining of liver metastatic lesions of PDAC tumor $Bmal1^{+/+}$ and $Bmal1^{-/-}$ mice with fibroblast markers: FAP (green); α-SMA (green); DESMIN (red); PDGFRα (green); and PDGFRβ (green).

H. Quantification of FAP^{+}, α-SMA^{+}, $DESMIN^{+}$, PDGFRα^{+} and PDGFRβ^{+} signals (n = 6 random fields per group).

I. Representative micrographs of immunohistochemical staining of lung metastatic lesions of CRC tumor in C57BL/6 wild type mice that received CRC tumor cell alone implantation, CRC tumor cell plus $Bmal1^{+/+}$ CAF co-injection and CRC tumor cell plus $Bmal1^{-/-}$ CAF co-injection with fibroblast markers: FAP (green); α-SMA (green); DESMIN (red); PDGFRα (green); and PDGFRβ (green).

J. Quantification of FAP^{+}, α-SMA^{+}, $DESMIN^{+}$, $PDGFR\alpha^{+}$ and $PDGFR\beta^{+}$ signals (n = 6 random fields per group).

K. Representative micrographs of immunohistochemical staining of lung metastatic lesions of PDAC tumor in C57BL/6 wild type mice that received PDAC tumor cell alone implantation, PDAC tumor cell plus $Bmal1^{+/+}$ CAF co-injection and PDAC tumor cell plus $Bmal1^{-/-}$ CAF co-injection with fibroblast markers: FAP (green); α-SMA (green); DESMIN (red) plus ENDOMUCIN (green); PDGFRα (green); and PDGFRβ (green).c

L. Quantification of FAP^{+}, α-SMA^{+}, $DESMIN^{+}$, $PDGFR\alpha^{+}$ and $PDGFR\beta^{+}$ signals (n = 6 random fields per group).

M. Representative micrographs of immunohistochemical staining of liver metastatic lesions of PDAC tumor in C57BL/6 wild type mice that received PDAC tumor cell alone implantation, PDAC tumor cell plus $Bmal1^{+/+}$ CAF co-injection and PDAC tumor cell plus $Bmal1^{-/-}$ CAF co-injection with fibroblast markers: FAP (green); α-SMA (green); DESMIN (red); PDGFRα (green); and PDGFRβ (green). Representative micrographs of immunohistochemical staining of lung metastatic lesions of

N. Quantification of FAP^{+}, α-SMA^{+}, $DESMIN^{+}$, $PDGFR\alpha^{+}$ and $PDGFR\beta^{+}$ signals (n = 6 random fields per group).

Data presented as mean determinants from random fields ± s.e.m. *$P < 0.05$; **$P < 0.01$; ***$P < 0.001$, ns = not significant; two-tailed student t-test and one-way ANOVA. Scale bar = 50 μm. meta = metastasis, TC = tumor cell

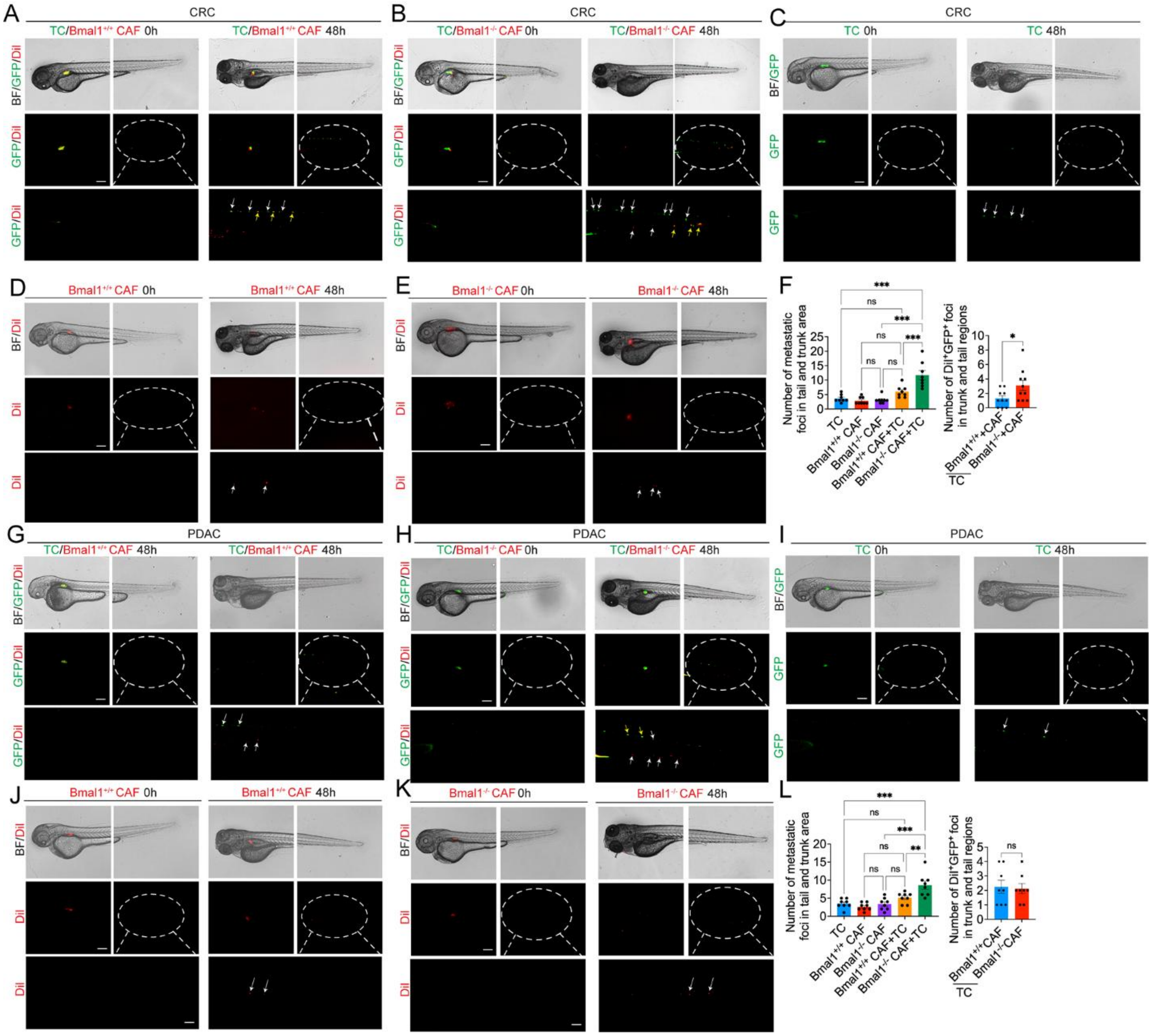

Supplemental Figure 8. Bmal1$^{-/-}$ CAFs promotes cancer metastasis in zebrafish.

A. Representative micrographs of zebrafish receiving co-implantation of GFP^{+} CRC cancer cells (green) plus DiI-labeled Bmal1$^{+/+}$ CRC-isolated CAFs (red) in the perivitelline space of each zebrafish embryo. At 48 h post-implantation, cancer metastasis was analyzed. White arrows indicate green or red cell clusters; yellow arrows point CAF-CRC (red merged green) cell clusters. Dashed lines encircle the amplified area of the trunk region (scale bars: 100 μm).

B. Representative micrographs of zebrafish receiving co-implantation of GFP^{+} CRC cancer cells (green) plus DiI-labeled Bmal1$^{-/-}$ CRC-isolated CAFs (red) in the perivitelline space of each

zebrafish embryo. At 48 h post implantation, cancer metastasis in was analyzed. White arrows indicate green or red cell clusters; yellow arrows point CAF-CRC (red merged green) cell clusters. Dashed lines encircle the amplified area of the trunk region (scale bars: 100 μm).

C. Representative micrographs of zebrafish receiving implantation of CRC cancer cells (green) in the perivitelline space of each zebrafish embryo. At 48 h post-implantation, cancer metastasis in was analyzed. White arrows indicate metastatic cancer cells. Dashed lines encircle the amplified area of the trunk region (scale bars: 100 μm; in amplified fields, 50 μm).

D. Representative micrographs of zebrafish receiving implantation of DiI-labeled Bmal1$^{+/+}$ CAFs (red) in the perivitelline space of each zebrafish embryo. At 48 h post-implantation, cell metastasis in was analyzed. White arrows indicate metastatic CRC-isolated CAF cells. Dashed lines encircle the amplified area of the trunk region (scale bars: 100 μm).

E. Representative micrographs of zebrafish receiving implantation of DiI-labeled Bmal1$^{-/-}$ CRC-isolated CAFs (red) in the perivitelline space of each zebrafish embryo. At 48 h post-implantation, cancer metastasis was analyzed. White arrows indicate metastatic CRC-isolated CAF cells. Dashed lines encircle the amplified area of the trunk region (scale bars: 100 μm).

F. Quantification of metastatic cells in the trunk and tail regions at 48 h post-implantation (n = 8 zebrafish embryos per group).

G. Representative micrographs of zebrafish receiving co-implantation of GFP^{+} PDAC cancer cells (green) plus DiI-labeled Bmal1$^{+/+}$ PDAC-isolated CAFs (red) in the perivitelline space of each zebrafish embryo. At 48 h post-implantation, cancer metastasis in was analyzed. White arrows indicate green or red cell clusters; yellow arrows point CAF-PDAC (red

merged green) cell clusters. Dashed lines encircle the amplified area of the trunk region (scale bars: 100 μm).

H. Representative micrographs of zebrafish receiving co-implantation of GFP^{+} PDAC cancer cells (green) plus DiI-labeled $Bmal1^{-/-}$ PDAC-isolated CAFs (red) in the perivitelline space of each zebrafish embryo. At 48 h post implantation, cancer metastasis in was analyzed. White arrows indicate green or red cell clusters; yellow arrows point CAF-PDAC (red merged green) cell clusters. Dashed lines encircle the amplified area of the trunk region (scale bars: 100 μm).

I. Representative micrographs of zebrafish receiving implantation of PDAC cancer cells (green) in the perivitelline space of each zebrafish embryo. At 48 h post-implantation, cancer metastasis in was analyzed. White arrows indicate metastatic cancer cells. Dashed lines encircle the amplified area of the trunk region (scale bars: 100 μm).

J. Representative micrographs of zebrafish receiving implantation of DiI-labeled $Bmal1^{+/+}$ PDAC-isolated CAFs (red) in the perivitelline space of each zebrafish embryo. At 48 h post-implantation, cancer metastasis in was analyzed. White arrows indicate metastatic PDAC-isolated CAF cells. Dashed lines encircle the amplified area of the trunk region (scale bars: 100 μm).

K. Representative micrographs of zebrafish receiving implantation of DiI-labeled $Bmal1^{-/-}$ PDAC-isolated CAFs (red) in the perivitelline space of each zebrafish embryo. At 48 h post-implantation, cancer metastasis in was analyzed. White arrows indicate metastatic PDAC-isolated CAF cells. Dashed lines encircle the amplified area of the trunk region (scale bars: 100 μm).

L. Quantification of metastatic cells in the trunk and tail regions at 48 h post-implantation ($n = 8$ zebrafish embryos per group).

Data presented as mean ± s.e.m. **P* < 0.05; ***P* < 0.01; ****P* < 0.001, ns = not significant; one-way ANOVA. Scale bar = 50 μm; TC = tumor cell.

Table S1. Primer sequences

Gene	Forward primer sequence	Reverse primer sequence
Actin	5'-AGGCCCAGAGCAAGAGAGG-3'	5'- AGGGTTGCACTAAACATGTCAG-3'
Serpine1	5'- GACACCCTCAGCATGTTCATC-3'	5'-GCCGTGTTAAGGAATCTGCTG-3'
Fap	5'- CACCTGATCGGCAATTTGTG-3'	5'-CCCATTCTGAAGGTCGTAGATGT-3'
Acta2	5'- ATTGTGCTGGACTCTGGAGATGGT-3'	5'- TGATGTCACGGACAATCTCACGCT-3'
Des	5'- CCTGGAGCGCAGAATCGAAT-3'	5'- TGAGTCAAGTCTGAAACCTTGGA -3'
Pdgfra	5'- TGGCATGATGGTCGATTCTA-3'	5'- CGCTGAGGTGGTAGAAGGAG -3'
Pdgfrb	5'- TCAACGACTCACCAGTGCTC -3'	5'- TTCAGAGGCAGGAAGGTGCT -3'
